# Aging-associated mechanisms of atrial fibrillation progression and their therapeutic potential

**DOI:** 10.20517/jca.2024.12

**Published:** 2024-11-07

**Authors:** Shuai Zhao, Aaron M. Johnston, Chi Him Kendrick Yiu, Lucia M. Moreira, Svetlana Reilly, Xander H. T. Wehrens

**Affiliations:** 1 Cardiovascular Research Institute, Baylor College of Medicine, Houston, TX 77030, USA.; 2 Department of Integrative Physiology, Baylor College of Medicine, Houston, TX 77030, USA.; 3 Division of Cardiovascular Medicine, Radcliffe Department of Medicine, University of Oxford, Oxford OX1 2JD, UK.; 4 Department of Pediatrics, Baylor College of Medicine, Houston, TX 77030, USA.; 5 Department of Medicine, Baylor College of Medicine, Houston, TX 77030, USA.; 6 Department of Neuroscience, Baylor College of Medicine, Houston, TX 77030, USA.; 7 Center for Space Medicine, Baylor College of Medicine, Houston, TX 77030, USA.

**Keywords:** Atrial fibrillation, arrhythmia, aging, myocardial remodeling, ryanodine receptor, therapeutic targets

## Abstract

Atrial fibrillation (AF) is the most common sustained arrhythmia, with a particularly high prevalence in the elderly. As the global aging population rapidly expands, it is increasingly important to examine how alterations to the aging heart contribute to an increased AF susceptibility. This work critically reviews the key molecular mechanisms that may underpin the complex association between aging and AF. Moreover, we identify emerging novel opportunities for therapeutic intervention that may be able to prevent and/or improve the current treatment paradigms for age-related AF. This review contributes to a holistic understanding of the intricate relationship between aging and AF.

## Graphical Abstract

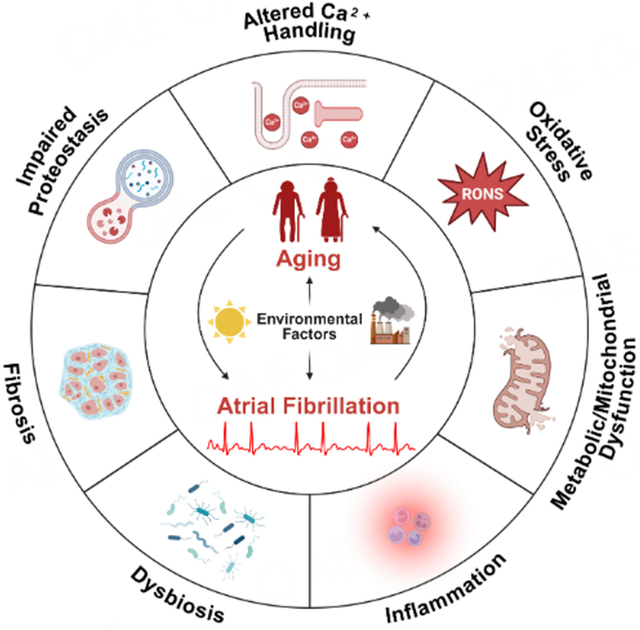

## INTRODUCTION

Atrial fibrillation (AF) is the most common, sustained cardiac arrhythmia. AF is associated with high mortality and morbidity, primarily driven by an increased risk of serious cardiovascular complications, such as stroke, heart failure, and sudden cardiac death. Globally, an estimated 60 million people experience AF^[1]^. The prevalence of AF has a marked age dependence: increasing from < 0.5% in people under 40 to > 5% in people older than 60 and reaching around 10% in people aged 80 or above^[2]^. These data position aging as a major risk factor for the development of AF. Owing to the significant successes of modern medicine, population aging is an increasingly pervasive and entrenched global phenomenon. In 2050, it is predicted that 16% of humans will be aged 65 or above, increasing from 10% in 2020^[3]^. In the context of this expanding aging population, it is crucial to identify and further elucidate the key molecular, cellular, and structural factors that contribute to the initiation, perpetuation, and progression of AF in the aging heart. In this pursuit, it is hoped that the first personalized preventive and/or curative pharmacotherapies for AF will be unveiled.

Aging can be defined as a progressive and irreversible decline in physiological integrity, driven by the accumulation of stressor-mediated damage and subsequent dysfunction at the cellular, tissue, and organ level^[4]^. Shared mechanisms, coined *Hallmarks of Aging*, have been identified and include genomic instability, telomere attrition, epigenetic alterations, loss of proteostasis, disabled macroautophagy, deregulated nutrient-sensing, mitochondrial dysfunction, cellular senescence, stem cell exhaustion, altered intercellular communication, chronic inflammation, and dysbiosis^[4,5]^. Rather than being an inevitable and precisely stereotyped decline, the aging process is plastic^[6]^; this raises the possibility that, with a more precise delineation of the mutual mechanisms of AF and aging, there may be opportunities to prevent and treat age-related conditions, including AF.

Age-related AF is a complex and multifactorial disease characterized by various pathophysiological changes in the atria including, but not limited to, altered calcium handling, oxidative stress, metabolic and mitochondrial dysfunction, inflammation and myocardial fibrosis, as well as emerging concepts such as dysbiosis of the microbiome and derailed proteolysis. Together, these changes, which are highly interconnected and themselves promoted by aging, constitute important mechanisms of AF pathogenesis, leading to decreased atrial function, maladaptive atrial remodeling, and ultimately, enhanced arrhythmia susceptibility. The diverse mechanisms outlined in this review exhibit strong associations with aging, thus highlighting the close connection between the disease and advancing age.

In this review, we provide an overview of various mechanisms underlying AF and how they may be instigated by aging. We emphasize the important molecular and cellular events that contribute to these processes, identifying key knowledge gaps, and speculate on future therapeutic strategies that may help uncouple advancing age from increased AF risk.

## OVERVIEW OF ARRHYTHMIA-PROMOTING MECHANISMS IN AF

In the early stages of the disease, AF is initiated by ectopic electrical activities outside the sinoatrial node, most commonly originating from the pulmonary veins, leading to paroxysms that result in irregular atrial activation rates faster than sinus rhythm^[7,8]^. As the disease progresses, the arrhythmia itself promotes further electrical, mechanical, and structural changes in the heart, leading to an increasingly persistent, electropathology-driven arrhythmia. This phenomenon is often termed “AF begets AF” and provides a mechanistic explanation as to why many interventions become less effective as the disease progresses^[8,9]^. The proarrhythmic changes include altered cardiomyocyte ion handling leading to early and delayed afterdepolarizations, shortening of the atrial effective refractory period, myocardial fibrosis causing conduction heterogeneity and impaired mechanical function, and inflammation.

Current treatment paradigms comprise three principal facets: Anticoagulation to reduce stroke risk; Better symptom control with rate and/or rhythm management strategies (both interventionally and pharmacologically); and Control of concomitant cardiovascular risk factors^[10]^. This *ABC* treatment approach predominantly aims to palliate the disease by managing symptoms and preventing cardiovascular complications without meaningfully striving to reverse, or even halt, progressive, structural myocardial remodeling. Moreover, antiarrhythmic pharmacotherapies themselves increase the risk of life-threatening arrhythmias^[11,12]^. Meanwhile, ablation and electrical cardioversion are associated with high (~20% and ~30%, respectively) AF recurrence rates^[13–15]^. A panacea for age-associated AF is likely to be developed through a sufficient understanding, and the subsequent targeted perturbation of the upstream, age-related arrhythmogenic remodeling processes.

## ALTERED CALCIUM HANDLING

Disruptions in Ca^2+^ homeostasis, typically emerging as a consequence of the other factors detailed below, contribute to AF initiation and maintenance^[16]^. Aging affects the regulation, storage and cycling of Ca^2+^ by altering the abundance and function of key Ca^2+^ handling proteins^[17]^. Altered Ca^2+^ handling changes cardiomyocyte electrophysiology (electrical remodeling), exacerbating the risk of delayed afterdepolarizations (DADs) and spontaneous atrial ectopic activity, both of which are key factors in AF initiation^[18]^.

**Figure 1.**
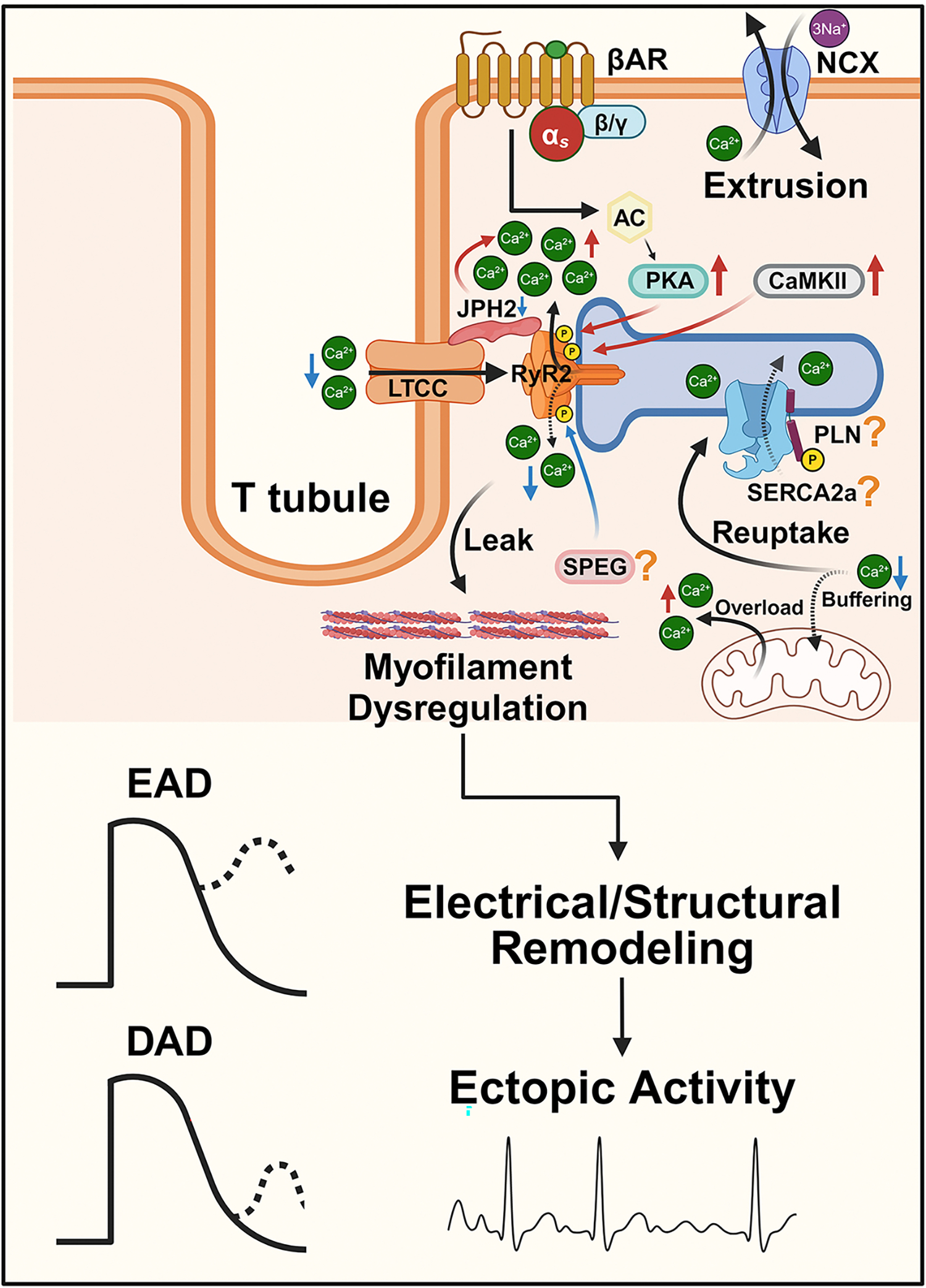
Altered calcium handling in aging and AF. In the aged heart, increased PKA and CaMKII signaling contributes to enhanced spontaneous SR Ca^2+^ release through hyperphosphorylation of RyR2. These alterations also contribute to myofilament dysregulation, thereby causing electrical and structural remodeling, cumulating in early and/or delayed afterdolarizations (EAD/DAD) during the action potential. SPEG phosphorylation of RyR2 inhibits diastolic Ca^2+^ leak, though its role in aging-related AF remains unclear (indicated by a question mark). JPH2 anchors RyR2 and LTCC. The level of JPH2 is decreased in the aged heart, while over-expression of JPH2 shows promise in stabilizing RyR2 and preventing SR Ca^2+^ leak, potentially reducing AF risk. The LTCC is reduced, leading to decreased peak Ca^2+^ concentration and a prolonged action potential duration (APD) in aged cardiomyocytes. Other calcium handling proteins include SERCA2a and PLN, whose expression levels and phosphorylation states remain debated in aged hearts (indicated by the question marks). Additionally, Mitochondrial dysfunction leads to impaired calcium buffering, resulting in cytosolic Ca^2+^ overload. JPH2: Junctophilin 2; LTCC: L-type calcium channel; PLN: phospholamban; RyR2: ryanodine receptor type 2; ROS: reactive oxygen species; SERCA2a: sarcoendoplasmic reticulum Calcium ATPase 2a; SR: sarcoplasmic reticulum. The figure was generated with BioRender.com.

In the aged heart, the sarco/endoplasmic reticulum Ca^2+^ ATPase type 2a (SERCA2a) efficiency has been suggested to be compromised [Figure 1]. Many studies suggest a reduction in its expression in aged hearts. For example, reduced SERCA2a levels were observed in myocardium tissue from mice over 20 months old compared to those younger than 6 months old, suggesting age-dependent decline^[19–21]^. However, this remains controversial, with some studies reporting no changes in SERCA expression. For example, one study revealed no relationship between age and the regulation of SERCA expression in human right atria^[22]^, while another research found no notable differences in SERCA2a expression between atrial and ventricular tissues of New Zealand rabbits aged 6 to 26 months^[23]^. Furthermore, concentrations of SERCA2a were found to be comparable between ventricular cardiomyocytes from young (2-month-old) and senescent (20–26-month-old) mice^[24]^. The differing findings may be explained by differences in species or nuances in experimental design. SERCA itself is regulated by its endogenous inhibitor phospholamban (PLN), whereby PLN phosphorylation status regulates its inhibitory effect on SERCA. While some studies have shown stable PLN phosphorylation levels in the myocardium of mice older than 2 years compared to those younger than 6 months^[19]^, other research observed a significant reduction in PLN phosphorylation in heart homogenates from 2-year-old rats relative to 6-month-old rats^[25]^. Again, these discrepancies may arise from species-specific differences in cardiac physiology^[19,25,26]^. In aged hearts, RyR2, the major SR Ca^2+^ release channel, becomes “leaky”^[26–28]^, contributing to increased spontaneous diastolic SR Ca^2+^ release. Increased PKA activity contributes to enhanced SR Ca^2+^ leak through hyperphosphorylation of RyR2 at PKA-dependent sites (Ser2808, Ser2030) [Figure 1]^[29,30]^. Additionally, Ca^2+^/calmodulin-dependent protein kinase II (CaMKII) phosphorylates RyR2, increasing its open state probability and leading to SR Ca^2+^ leak through RyR2 [Figure 1]^[31,32]^. However, not every RyR2 phosphorylation site increases its activity. RyR2 phosphorylation at Ser2367 by SPEG, the expression of which is reduced in human paroxysmal AF, reduces diastolic Ca^2+^ leak [Figure 1]. Whether reduced SPEG activity is also a proarrhythmic feature during aging is unexplored but warrants further investigation^[33]^.

Dysfunctional Ca^2+^ release from the SR leads to a significant increase in the cytosolic Ca^2+^ concentration, establishing a proarrhythmic environment. Notably, increased myocardial CaMKII signaling is associated with aging and is observed in old mice (104 weeks)^[34]^. However, it is not only RyR2 and CaMKII that exhibit age-related changes. Junctophilin-2 (JPH2) anchors L-type Ca^2+^ channels (LTCCs) and RyR2 in close proximity, facilitating the efficient activation of RyR2 by Ca^2+^ ions entering the cardiomyocyte through LTCCs [Figure 1]^[35]^. Impaired JPH2-mediated regulation of RyR2 has been implicated in arrhythmogenesis^[36]^, with downregulation of JPH2 leading to elevated Ca^2+^ release event frequency, while its overexpression reduces event frequency^[37,38]^. The recently reported findings that JPH2 improves age-associated remodeling of excitation-contraction coupling in heart failure further underscore the role of JPH2 in addressing impaired structural and functional regulation in the aging heart^[39]^. It is possible that targeting JPH2 may also offer a promising therapeutic approach for aging-associated AF. The L-type Ca^2+^ current, a crucial component of the action potential (AP), is reduced^[40]^ and the action potential duration (APD) is increased in aged cardiomyocytes [Figure 1]^[41,42]^. These alterations in Ca^2+^ homeostasis affect the kinetics of Ca^2+^ transients. The peak Ca^2+^ concentration during systole shows a tendency to decrease with age, which is associated with a reduced Ca^2+^ transient amplitude. These alterations are accompanied by a prolonged time to the peak and decay of Ca^2+^ levels^[26,43]^, favoring the decline of contractile function. Interestingly, studies have reported that the density and expression of LTCC remain constant in aged heart, while the transmembrane Ca^2+^ currents are affected due to other age-related changes, such as expanded cell membrane area. This observation was mainly made in ventricular cardiomyocytes, and further research is needed to determine whether similar effects occur in atrial cardiomyocytes^[23,44]^. Mitochondria also play a role in Ca^2+^ regulation by sequestering and buffering Ca^2+^. Mitochondrial dysfunction has been associated with age, leading to a reduced ability to buffer Ca^2+^, which further exacerbates cytosolic Ca^2+^ overload associated with AF [Figure 1]^[45,46]^. The combined impact of these age-related changes in Ca^2+^ handing proteins [Figure 1], often precipitated by upstream changes that are detailed below, results in functional alterations that predispose the aging heart to AF.

## OXIDATIVE STRESS

Oxidative stress (OS), a state in which the production of radical and non-radical reactive oxygen and nitrogen species (RONS) preponderates over the cell’s endogenous antioxidative defense capacity, has been proposed as one mechanism of age-associated cellular damage^[47,48]^. Cellular dysfunction emerges following the accumulation of oxidative damage to macromolecules (DNA, proteins, and lipids), culminating in cell senescence and inflammation, coined oxi-inflamm-aging^[49,50]^. Aging promotes increased RONS production through the accumulation of mitochondrial DNA mutations and subsequent mitochondrial dysfunction (further enhancing ROS production) and chronic systemic inflammation alongside a decline in antioxidant abundance^[51,52]^. Moreover, longer lifespans are often accompanied by increased exposure to environmental pro-oxidants, e.g., smoking, air pollution, and UV light^[53]^.

### Oxidative stress in AF pathogenesis

**Figure 2.**
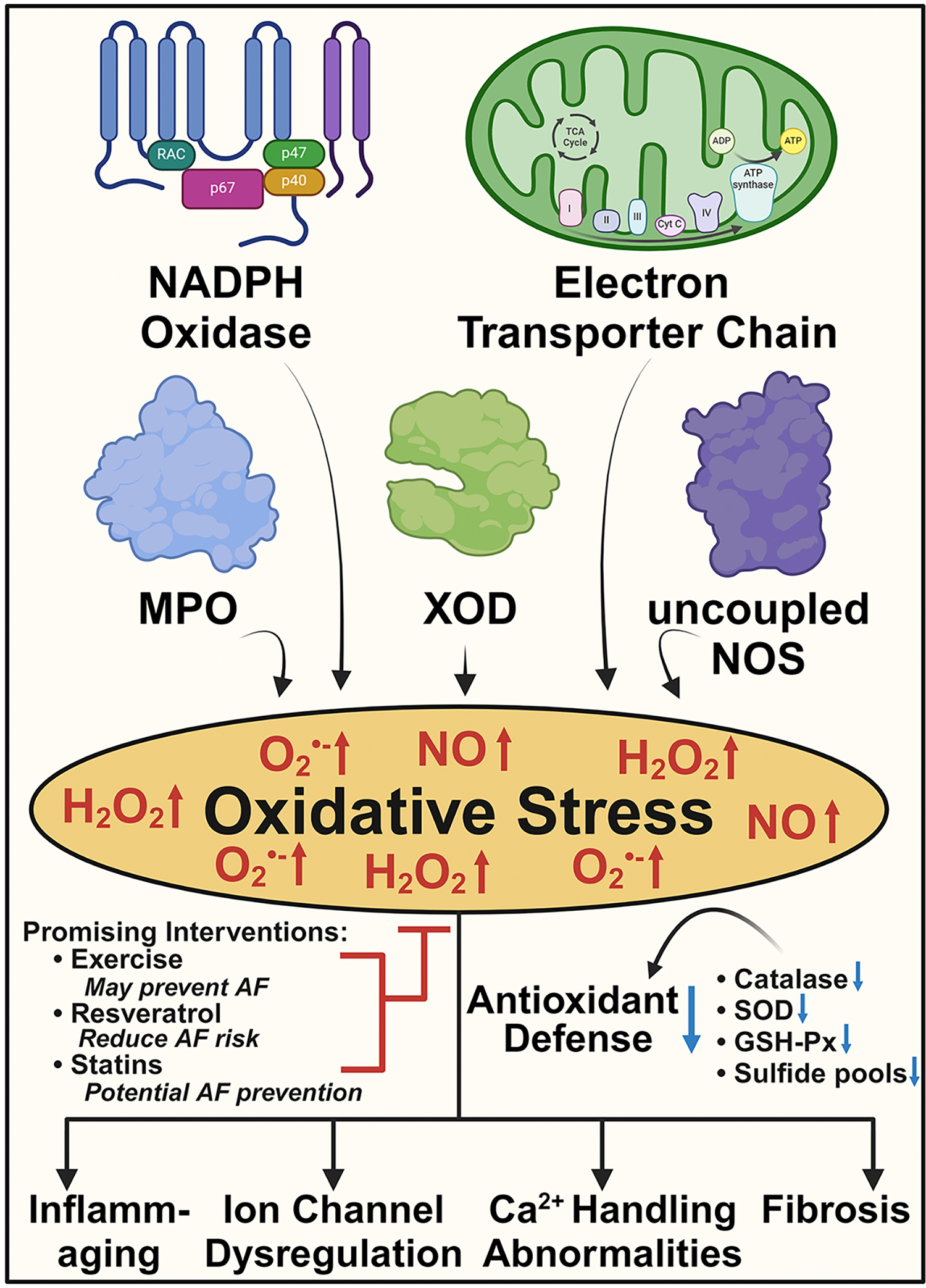
Oxidative stress in aging and AF. Imbalance between reactive oxygen and nitrogen species (RONS) production by NADPH oxidase, myeloperoxidase (MPO), xanthine oxidase (XOD), uncoupled nitric oxide synthase (NOS), and the mitochondrial electron transport chain causes oxidative stress, contributing to age-related cellular damage and AF. Dysregulation of acid-labile sulfide pools, catalase, superoxide dismutase (SOD), and glutathione peroxidase (GSH-Px) in aged hearts worsen this imbalance. Exercise, resveratrol, and statins increase antioxidant defenses and show promise in reducing the risk of age-related AF in preclinical or clinical studies, pending further validation through larger-scale studies. The figure was generated with BioRender.com.

The heart, with its significant metabolic demand, is particularly vulnerable to OS and is thus an area of intense research in the field of age-associated AF pathogenesis^[54]^. Atrial RONS sources include the NADPH oxidase, xanthine oxidase, uncoupled nitric oxide synthase, myeloperoxidase (MPO), and the mitochondrial electron transport chain. [Figure 2] NADPH oxidase, as the major source of the highly reactive radical superoxide anion (O_2_^•−^), has been implicated in AF pathogenesis, particularly in the early stages of disease^[55]^. Similarly, mitochondrial oxidases, uncoupled nitric oxide synthases (NOS), and MPO have also been shown to play a pathological role at different stages of AF progression^[55,56]^. Reactive oxygen/nitrogen species, through modification of ion channels, Ca^2+^ handling protein activity, and the potentiation of inflammation, contribute to a permissive electrical and structural substrate and have been linked to AF pathogenesis^[57,58]^.

There is also evidence that antioxidant defense and RONS scavenging capacity are reduced both in AF and with aging. Antioxidant enzymes primarily include cytoplasmic and mitochondrial superoxide dismutase (SOD), catalase, glutathione peroxidase (GSH-Px), and glutaredoxins^[59]^. Indeed, circulating levels of manganese SOD are lower in patients with paroxysmal AF *vs.* control or persistent AF and there is reduced expression of GSH-Px in the right atrial appendages of patients with AF compared to sinus rhythm controls^[60,61]^. Similarly, the enzymatic antioxidant defense is impaired with age, as evidenced by a significant reduction in the activity of SOD-1, catalase and GSH-Px in erythrocytes [Figure 2]^[62]^.

Furthermore, changes in non-enzymatic antioxidants in AF have been reported. For example, plasma acid labile sulfide pools, from which the antioxidant gasotransmitter H_2_S can be generated, are reduced in human AF, while administration of a sulfide donor (diallyl trisulfide) decreased inducible AF incidence in mice lacking the key H_2_S-producing enzyme (cystathionine-γ-lyase, CSE)^[63]^. Intriguingly, endothelial-specific CSE-overexpression in the context of global CSE deficiency alone prevented AF, which links atrial remodeling and AF to vascular endothelial dysfunction, a hallmark of the vascular aging process^[63,64]^. Notably, the AF subjects in this study were significantly older than the control group, which leaves the possibility that the differences in H_2_S/sulfides were due to advancing age rather than AF. Nevertheless, the existence of an AF-promoting, pathogenic crosstalk between endothelium and a vulnerable myocardium in the presence of OS in the aging heart warrants further exploration.

### Therapeutic targeting of oxidative stress in AF

The therapeutic utility of antioxidative interventions in AF has been explored. For example, the flavonoid polyphenol resveratrol, an antioxidant and anti-inflammatory derivative of stilbene, has been shown to have antiarrhythmic effects [Figure 2]. In the context of heart failure or hypertrophy, resveratrol was able to normalize ion channel activity and Ca^2+^ handling, suppress myocardial fibrosis, and importantly, reduce AF susceptibility^[65,66]^. As these mechanisms are pertinent to the pathogenesis of AF, the putative benefits of pure resveratrol, or its derivatives with improved pharmacokinetic parameters, in AF prevention and/or treatment warrant interrogation in human clinical trials^[67,68]^. Alternative antioxidant strategies, such as molecular hydrogen-enriched saline, have been shown to mitigate AF incidence and duration, alleviate atrial fibrosis, and inhibit the activity of the JAK/STAT3 pathway in a rat model of pressure overload cardiac hypertrophy^[69]^. Similarly, the antioxidants ascorbic acid (vitamin C) and N-acetylcysteine (NAC) have been shown to be effective in the prevention of postoperative AF. Further large-scale trials are required to assess whether antioxidant supplementation can prevent age-related AF.

### Oxidative stress in AF: cause or consequence?

However, despite reports of enhanced oxidative stress in AF, aspersions have been cast on its causal role in AF pathogenesis. Recently, it was shown that atrial overexpression of gp91^phox^-containing NAPDH oxidases (NOX2), the activity of which is upregulated in the atria in AF and represents the predominant sources of atrial O_2_^•−^, does not contribute to atrial electrical or structural remodeling parameters in mice and has modest effects on AF inducibility but no effect on AF duration^[70,71]^. These data challenge the causal role of NOX2-derived superoxide (O_2_^•−^) in AF causation or progression in rodents and instead suggest enhanced superoxide levels may represent a biomarker of AF susceptibility, rather than a causal contributor to AF risk. Although NADPH-stimulated O_2_^•−^ production was enhanced, direct evidence of increased oxidative stress in the unstimulated myocardium was not shown, suggesting the possibility of compensation from increased O_2_^•−^ buffering capacity. Similarly, whether other myocardial sources of RONS play a causative role in AF cannot be excluded.

Data from human studies have also failed to definitively define the role of oxidative stress in age-related AF pathogenesis. Statins inhibit Rac1 activity to reduce superoxide production and were shown to be protective against AF in preclinical models [Figure 2]^[72,73]^. However, in the randomized, placebo-controlled Statin Therapy in Cardiac Surgery (STICS) trial, perioperative rosuvastatin therapy failed to prevent postoperative AF^[74]^. These results come with the caveat that the etiology of postoperative AF, or AF induced by rapid pacing protocols in rodents, differs from lone, age-related AF^[74]^. Given that earlier animal studies showed promise for statins in AF prevention, these results also highlight the importance of considering species-specific biological differences. Furthermore, rosuvastatin is a hydrophilic statin that confers strong hepatoselectivity but less capacity for extra-hepatic, pleiotropic effects, such as inhibiting myocardial RONS sources^[75]^. Additionally, the STICS trial participants were relatively young (mean age: 59) and were mostly statin-naïve. Yet, the results of meta-analyses have not found evidence that statin therapy prevents AF^[76]^. The discrepant results signify the importance of deploying combinatorial approaches in future research to faithfully capture the complexity of AF pathogenesis. The continuing identification and clinical efficacy of more potent and cardiac-specific antioxidant therapies may provide new therapeutic avenues for age-associated AF.

### Physical activity and AF

Alongside pharmacological interventions, lifestyle modifications could reduce the risk of age-related AF [Figure 2]. In the older population, both moderate aerobic and resistance exercise increases antioxidant reserves, reduces oxidative damage, and suppresses systemic inflammation^[47,77]^. Encouragingly, data from the prospective Cardiovascular Health Study demonstrated that in older adults, moderate exercise, compared to no exercise, was associated with a 28% reduction in AF incidence^[78]^. Although the design of this study may have missed episodes of paroxysmal AF and relied on inherently subjective self-reported activity assessments, it implies holistic lifestyle interventions could prevent up to a quarter of incident AF cases among the elderly. Exercise interventions, lacking the traditional side effects associated with pharmacotherapies, are increasingly studied for the treatment and prevention of AF, but their efficacy and optimal design, specifically in elderly cohorts, require further study^[79]^. The relationship between aging and AF with oxidative stress is summarized in Figure 2.

## ALTERED METABOLISM IN AF

In AF, rapid activation rates establish a hypermetabolic rate, and transcriptomic and proteomic studies provide evidence of upregulated glycolytic pathways and ketone body metabolism in persistent AF^[80,81]^. Moreover, as metabolic changes have also been observed in tissue samples acquired prior to the onset of postoperative AF, these changes may contribute to AF onset rather than arising only as a consequence of AF^[81]^. Additionally, a recent study identified unique profiles of myocardial amino acid metabolites (e.g., reduced alanine levels) between aged (13-month-old) *vs.* adult (2–3-month-old) mice with experimental AF. These metabolic changes cannot be accounted for by advanced age alone as they were not present in aged (13-month-old), sinus rhythm controls^[82]^. In humans, age-related AF was also associated with altered plasma amino acid metabolites^[82]^. These results imply age-related AF is associated with its own unique metabolomic profile, which is distinct from early-onset AF and not due to aging *per se*. Further studies should aim to establish whether these metabolomic changes are causal in AF development and whether dietary interventions to restore metabolomic homeostasis could be a viable therapeutic strategy.

## MITOCHONDRIAL DYSFUNCTION IN AF

**Figure 3.**
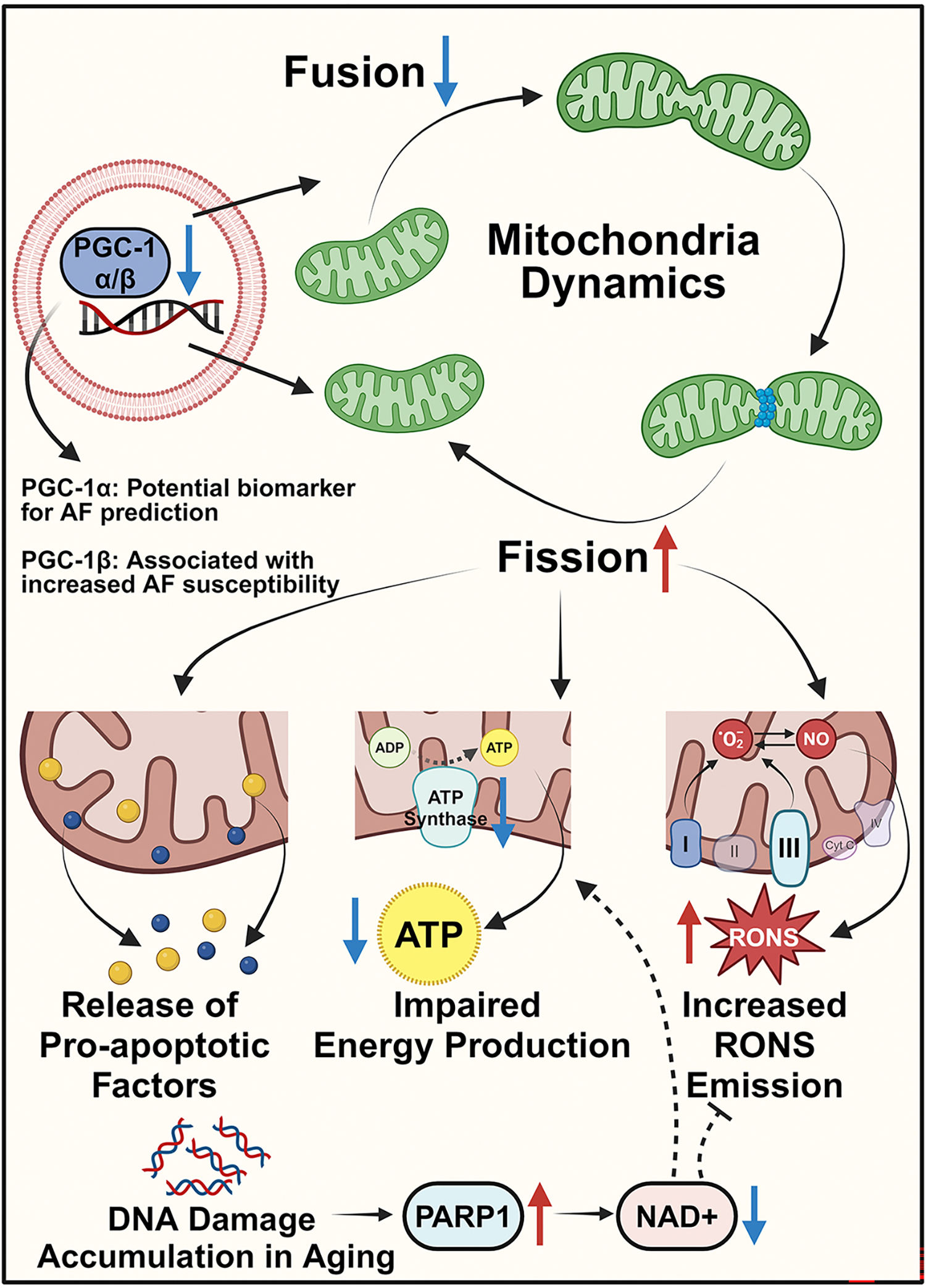
Mitochondrial dysregulation in aging and AF. Dysregulation of PGC-1α and PGC-1β during aging affects mitochondrial biogenesis, which results in enhanced cell death, impaired energy production, and increased RONS levels, promoting an age-dependent atrial arrhythmic phenotype. PGC-1α may serve as a potential biomarker for AF prediction, while reduced PGC-1β is associated with an increased risk of age-related arrhythmias. Mitochondrial complexes, particularly I and III, contribute to superoxide (O_2_^•−^) production, which interacts with nitric oxide (NO) to form RONS. Accumulated DNA damage during aging activates PARP, which depletes NAD+, further dampening ATP generation and exacerbating oxidative stress. NAD: Nicotinamide adenine dinucleotide; PGC: peroxisome proliferator-activated receptor gamma coactivator; PARP: poly (ADP-ribose) polymerase; RONS: reactive oxygen and nitrogen species. The figure was generated with BioRender.com.

Mitochondria are membrane-bound organelles present in eukaryotic cells and occupy, by volume, 1/3rd of the myocardial space^[83]^. Mitochondria are responsible for 95% of ATP generation, underscoring their critical role in coupling myocardial energy demand with metabolic activity^[84]^. RONS are generated as a byproduct of mitochondrial metabolism [Figure 3], and as AF progresses, the mitochondrial electron transport chain, alongside nitric oxide synthase (NOS), becomes the predominant source of RONS^[55]^. The impairment of bioenergetic mitochondrial function can result from various factors, including age-related damage, the accumulation of somatic mitochondria DNA mutations, and enhanced oxidative damage^[85,86]^. Therefore, mitochondrial dysfunction has been established as one of the key hallmarks of aging^[87]^ and is linked to the development of numerous age-related disorders, in both humans and experimental models of AF^[46]^. The involvement of mitochondria abnormality in AF was first proposed in the 1970s, when a decreased abundance and quality of mitochondria was observed in AF patients^[88]^. Decades later, disrupted mitochondrial structure and function was reported in experimental models of AF^[89]^. Since then, a growing body of evidence has emerged to support the role of mitochondria dysfunction, characterized by altered ATP production, loss of mitochondrial membrane potential (Ψ_mito_), and fragmented mitochondrial ultrastructure [Figure 3], as a contributor to AF in a diverse range of model systems and clinical samples^[90]^. However, there remains debate as to whether AF drives mitochondrial dysfunction, or vice versa, or whether the two processes mutually reinforce to culminate in an increasingly obstinate disease. To more conclusively define the causal role of mitochondrial dysfunction, changes in mitochondrial function should be demonstrated prior to the onset of spontaneous AF, which can be reversed/prevented by improving mitochondrial function.

Peroxisome proliferator-activated receptor gamma coactivator 1 alpha (PGC-1α) is a key transcriptional coactivator that regulates mitochondrial biogenesis and function in heart tissue^[91,91]^. A clinical trial found that elderly AF patients exhibited significantly reduced levels of serum PGC-1α, indicating its potential as a novel predictive biomarker for age-related AF [Figure 3]^[46]^. Interestingly, PGC-1β- another member of the PGC family - also contributes to mitochondrial dysfunction and an age-dependent atrial arrhythmic phenotype in mice [Figure 3]^[92]^. Homozygous *Pgc-1β*-deficient mice possess a pronounced mitochondrial defect with older mice displaying abnormal action potentials, augmented fibrotic remodeling in the heart, and a greater propensity for arrhythmias compared to younger (3 months) mice^[92]^. Furthermore, in a knock-in murine model of a gain-of-function RyR2 mutation leading to intracellular calcium leak, mice exhibited age-related atrial RyR2 oxidation, mitochondrial dysfunction, and increased reactive oxygen species (ROS) production, causing age-dependent increased susceptibility to AF^[58]^. Meanwhile, genetic inhibition of mitochondria ROS effectively prevented AF in these transgenic mice^[58]^. Studies of this kind demonstrate the interconnectedness of age-related AF pathomechanisms.

Considering the rapid atrial activation in AF, the already considerable metabolic demand of the myocardium is further enhanced^[93]^. NAD+, the precursor of the major source of the reductive power for RONS detoxification, NADPH, is reduced in tachypaced HL-1 cardiomyocytes due to poly(ADP-ribose) polymerase 1 (PARP1) activation^[94]^. PARP1, a nuclear enzyme that carries out protein poly(ADP-ribosyl)ation, is upregulated by DNA damage, as is accumulated during aging, and acts as a caretaker of genome stability to promote longevity^[95]^. However, PARP1 activation also depletes NAD+, a major player in oxidative defense, which exacerbates oxidative damage [Figure 3]. Therapeutically boosting NAD+ levels has been shown to improve age-related ischemic conditions and is being investigated in a number of clinical trials for a diverse range of diseases^[96,97]^. Indeed, the HF-AF ENERGY trial aims to assess whether nicotinamide riboside, an NAD+ precursor shown to be safe and to boost NAD+ levels in humans, can normalize blood-based markers of mitochondrial function and the NAD metabolome and, crucially, AF burden in the context of ischemic cardiomyopathy^[98,99]^. Studies like these might offer insight into the role of dysregulated PARP1-NAD+ signaling in aging and AF and, ultimately, provide innovative strategies for the therapeutic targeting of mitochondrial dysfunction and DNA damage to reduce the burden of age-related AF. The relationship between aging and AF in mitochondria dysfunction is summarized in Figure 3.

## INFLAMMATION

**Figure 4.**
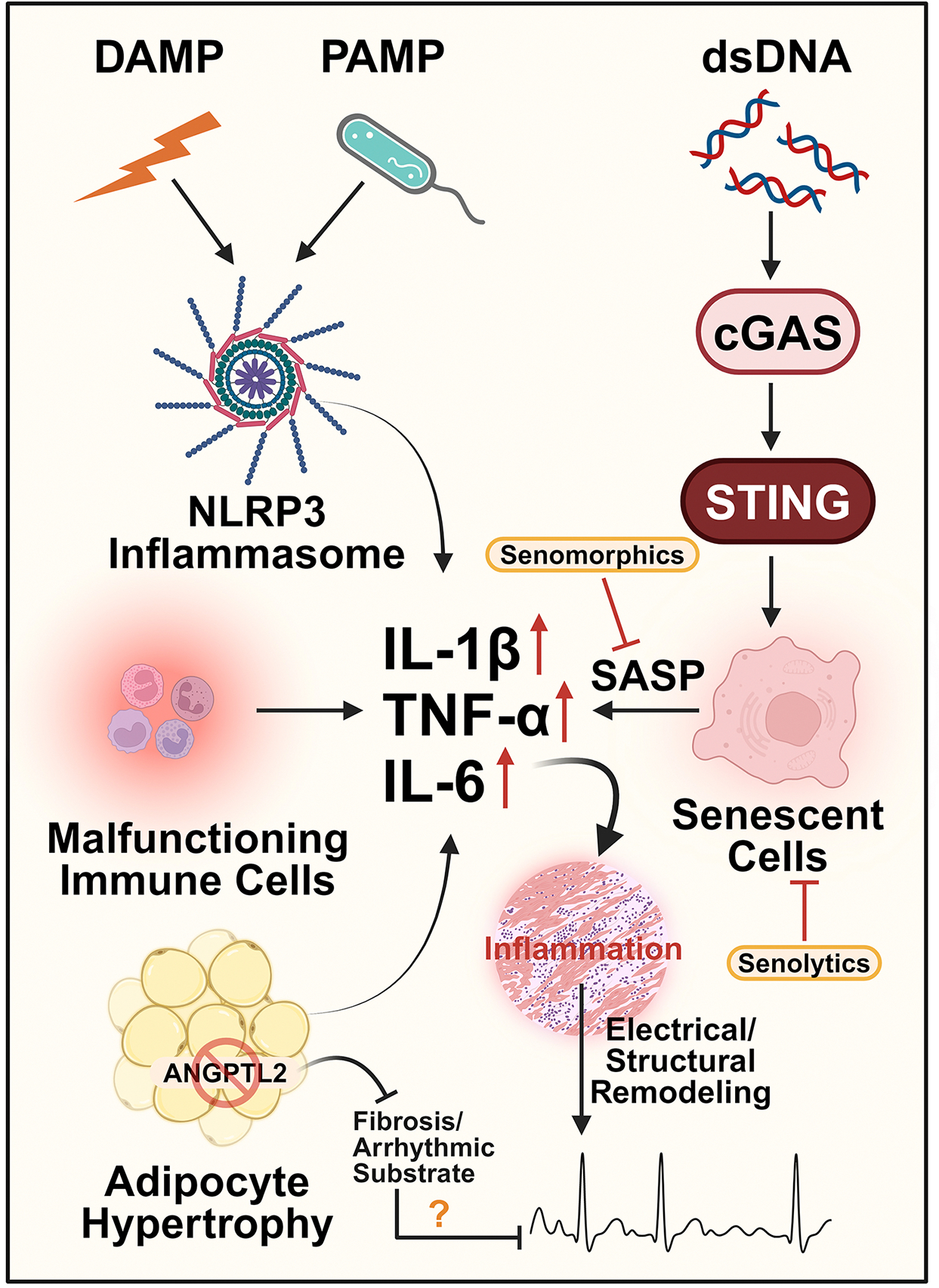
Inflammation in aging and AF. Aging causes adipocyte hypertrophy and malfunction in immune cells. The production of damage-associated molecular patterns (DAMPs) and pathogen-associated molecular patterns (PAMPs) causes activation of the NLRP3 inflammasome. The release of double-stranded DNA (dsDNA) into the cytosolic activates the cGAS-STING signaling pathway, although there is currently no direct evidence implicating this pathway in aging-related AF. Activation of cGAS leads to the development of the senescence-associated secretory phenotype (SASP) and recruitment of immune cells to eliminate damaged and senescent cells. These processes lead to increased expression of proinflammatory cytokines such as IL-1β, IL-6, and TNF-*α*. The elevated cytokine levels result in chronic inflammation within the heart, which can promote ectopic activity and the formation of substrates that trigger AF. Targeting inflammation by reducing senescence with senolytics and senomorphics and addressing the inflammatory role of epicardial adipose tissue (EAT) with ANGPTL2-targeting therapies may offer potential therapies for managing age-related AF. The figure was generated with BioRender.com.

Systemic inflammation occurs as part of the aging process, termed “*inflammaging*”. Chronic, physiological stimulation of the innate immune system drives inflammaging and the persistent production of inflammatory cytokines such as IL-1, IL-6, TNFα, and CRP [Figure 4], all of which have been shown to be involved in the pathogenesis of various age-related diseases^[100]^. It is noteworthy that inflammaging is caused by heterogeneous, but interconnected, mechanisms, including cellular senescence, inflammasome activation, oxidative stress, mitochondrial dysfunction, immune cell dysregulation, chronic infections, and dysregulated autophagy-lysosomal systems^[101]^.

Inflammation has also long been suspected to play a role in AF pathogenesis. For instance, the levels of the systemic inflammation marker C-reactive protein (CRP) are significantly elevated in AF patients^[102]^. AF also contributes to inflammation, perpetuating an inflammatory state and establishing a prothrombotic environment^[103]^. There is now growing appreciation that inflammation serves as an important driver of AF and its associated electrical and structural remodeling^[104–107]^. Chronic inflammation in aging promotes myocardial fibrosis and disrupts ion channel function and intracellular Ca^2+^ handling^[108]^.

### Cell senescence and AF

Cell senescence, a response to acute or chronic cellular damage, plays a central role in inflammaging and is recognized as a hallmark of aging. Cell senescence is typically initiated by telomere shortening and the accumulation of genotoxic damage leading to proliferative arrest^[109]^. Senescent cells adopt a senescence-associated secretory phenotype (SASP), secreting cytokines and chemokines, which contribute to inflammaging and progressive tissue fibrosis [Figure 4]^[109]^. The use of senolytics to pharmacologically eliminate senescent cells, and senomorphics to suppress the SASP, has demonstrated promise in the treatment of age-associated cardiovascular diseases in preclinical models [Figure 4]^[110]^. Interestingly, some drugs traditionally used in AF management, such as digoxin, have senolytic activity *in vitro* and *in vivo*^[111]^. Notably, in recent years, the cyclic GMP-AMP synthase (cGAS)-stimulator of interferon genes (STING) pathway, activated by the leakage of double-stranded DNA molecules, has emerged as an important link between senescence and inflammaging by contributing to the SASP [Figure 4]^[112,113]^. Thus, blockade of cGAS-STING signaling could ameliorate inflammatory responses with aging and prevent AF progression. However, anti-senolytics must be deployed cautiously in aging populations, as senescence and the SASP play important roles in tissue homeostasis, tumor suppression, and the resolution of fibrosis and wound healing^[114]^. Establishing cardiac-specific senolytics/senomorphics and determining the most suitable cohorts and timing for therapeutic intervention are remaining obstacles on the journey to clinical adoption of senolytics for AF.

### Inflammasome activation and AF

The contribution of inflammasome activation to inflammaging has drawn widespread attention [Figure 4]^[115,116]^. Meanwhile, various studies have begun to elucidate the role of inflammasome signaling in AF pathogenesis, with a particular focus on the activation of the NLRP3 inflammasome^[117–119]^. The inflammatory signaling regulated by the NLRP3 inflammasome promotes abnormal SR Ca^2+^ release and electrical remodeling in the atria of CM-specific knock-in mice expressing constitutively active NLRP3^[119]^. Results from fecal matter transplants (detailed further below) have demonstrated that age-related AF susceptibility is transferrable and associated with increased activation of the atrial NLRP3 inflammasome^[118]^. Another study examining 24-month-old NLRP3 knockout mice found that NLRP3 deficiency improved cardiac function by preventing metabolic dysregulation and mitigating other negative effects of cardiac aging^[120]^. Additionally, NLRP3 deficiency reduced cardiac damage by protecting the prolongation of the age-dependent PR interval, which is associated with AF^[120]^. Although pharmacologically targeting the NLRP3 inflammasome is still at an early stage, the causal relationship between inflammasome activation and AF identified in preclinical studies emphasizes the need for clinical research into the potential therapeutic benefits of NLRP3 inhibition in age-related AF. Importantly, atrial NLRP3 inflammasome activation typically triggers fibrosis by amplifying inflammatory factor secretion and fibrosis-related protein deposition^[118,119]^.

### Crosstalk between adipose tissue and the myocardium in AF

Aging also induces hypertrophy of epicardial adipose tissue (EAT), promoting an enhanced inflammatory secretome profile that transmits inflammation to the adjacent myocardium [Figure 4]. This inflammatory micro-environment contributes to adverse ion channel and connexin expression, as well as fibrotic remodeling, thereby increasing AF susceptibility^[121,122]^. Therapeutically targeting EAT or its related components might represent a novel intervention to combat AF. For example, angiopoietin-line protein 2 (ANGPTL2) in EAT was shown to positively correlate with atrial fibrosis in AF patients^[123]^, while pharmacological inhibition of Angptl2 significantly reduced fibrotic gene expression in primary rat cardiac fibroblasts and ameliorated induced fibrosis in organo-cultured rat atria [Figure 4]^[124]^. The encouraging finding suggests the potential efficacy of targeting ANGPTL2 in EAT to prevent age-related AF in the future. Another study found that the EAT secretome of AF patients had proinflammatory and profibrotic effects that intensified with AF progression, and that this EAT was enriched with neutrophils, which secrete numerous profibrotic molecules such as myeloperoxidase^[125]^. Targeting these molecules could be considered in future therapeutic interventions for age-related AF. The relationship between aging and AF in inflammation is summarized in Figure 4.

## DYSBIOSIS OF THE MICROBIOME

**Figure 5.**
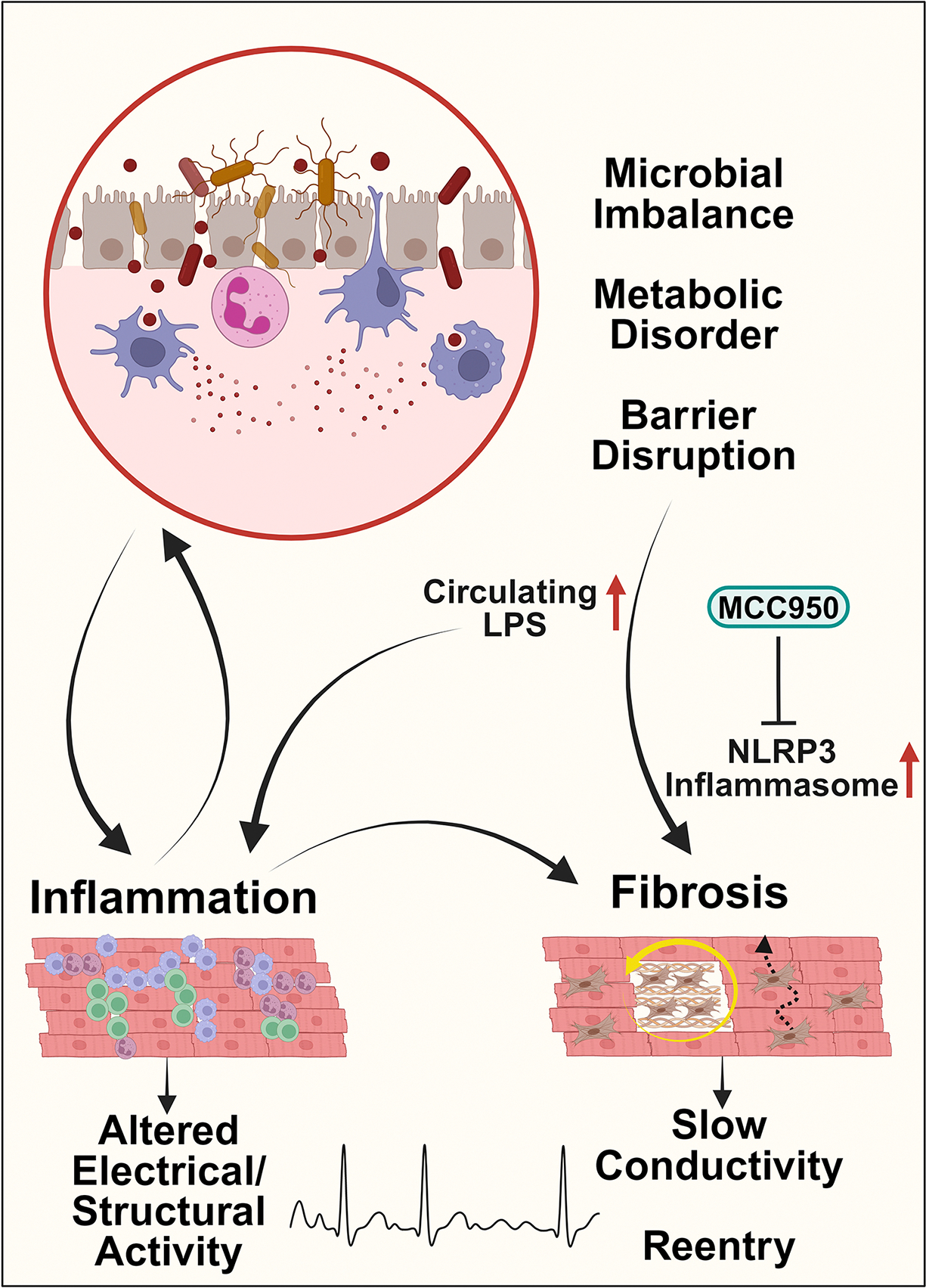
Dysbiosis in aging and AF. Aging-associated dysbiosis causes gut barrier impairment and an imbalance in gut microbe-derived metabolites, which causes increased levels of circulating lipopolysaccharide (LPS). LPS stimulates the secretion of inflammatory cytokines and activates the NLRP3 inflammasome in aged atria, exacerbating atrial fibrosis. This inflammatory environment facilitates the formation of fibrosis, leading to slowed conduction and reentrant arrhythmias. Inflammation in AF can also aggravate dysbiosis by disrupting gut microbiota balance, which worsens gut dysbiosis and perpetuates inflammation in the heart. Notably, NLRP3 inhibitor MCC950 has been shown to decrease fibrosis in atria and reduce AF susceptibility. The figure was generated with BioRender.com.

The microbiota comprises 10–100 trillion commensal, symbiotic and pathogenic microorganisms that inhabit our body surfaces, most abundantly in the gut, which regulate their host’s organ function^[126]^. These organisms play essential roles in host defense, nutrient absorption, and metabolite production. Dysbiosis, characterized by a decrease in the diversity of microbiota species and a shift toward a more proinflammatory architecture, is increasingly appreciated as a contributor to the pathogenesis of many age-related diseases [Figure 5]^[4,127]^. Large clinical studies, validated in independent cohorts, have shown that the aging microbiota becomes increasingly ecologically unique in older individuals who retain good health, whereas a lower uniqueness and an increased relative abundance of the *Bacteroides* genus are associated with higher mortality in those over 85 years of age^[128]^. These findings indicate that the composition of the microbiota, and the metabolites they produce, in the aging human may shape the trajectory of their health.

Given that age and many other established AF risk factors (e.g., sex, obesity, hypertension, heart failure) are associated with changes in the composition and function of the microbiome, recent studies have examined whether microbiome alterations affect AF risk and progression^[129]^. Zhang *et al.* showed that fecal microbiota transplanted from aged (22–24 months old) rats transfers the higher AF susceptibility to younger rats (2–3 months old)^[118]^. Young rats that acquired fecal microbiota showed increased levels of circulating lipopolysaccharide (LPS) and glucose, as well as atrial NLRP3 inflammasome activation and increased atrial fibrosis [Figure 5]^[118]^. Treatment with MCC950, a selective inhibitor of NLRP3, ameliorated atrial fibrosis and reduced AF susceptibility [Figure 5]^[118]^. Furthermore, microbiota-derived metabolites, such as trimethyl amine-N-oxide, can promote arrhythmias in canines when injected into the atrial ganglionic plexi and can aid AF risk prediction in elderly populations, independently of traditional risk factors^[130,131]^.

Observational studies have identified dysbiosis and altered fecal and serum metabolites in patients with persistent AF, regardless of its duration, suggesting dysbiosis may be involved early in AF initiation and/or progression^[132]^. Subsequently, a much larger study correlated prevalent AF with changes in nine genera, without change in overall microbiome diversity^[133]^. However, in both studies, the existing AF participants were significantly older than the controls without AF. Moreover, the microbiome changes associated with incident AF were largely inconsistent with those associated with existing AF, with the exception of the changes in the *Enorma* genus^[133]^. The most dysregulated genera were also inconsistent between these studies, which may be due to different population ethnicities, drug/lifestyle exposures, or AF heterogeneity^[132,133]^.

Prospective, interventional studies are needed to untangle whether dysbiosis is a cause or consequence of AF and whether distinct enterotypes may contribute to AF initiation *vs.* maintenance. Additionally, mechanistic insight into how microbiome alterations may promote the proarrhythmic substrate in the aging heart is limited yet is required for rational design of potential antiarrhythmic, microbiome-modifying interventions for elderly patients, e.g., pre/pro-biotics, fecal microbiota transplants, and diet interventions^[129]^. Such work should ultimately aim to identify an antiarrhythmic enterotype, and its associated metabolome, along with the modifiable factors that establish it. The relationship between aging and AF in dysbiosis is summarized in Figure 5.

## FIBROSIS

### Cardiac fibrosis and aging

**Figure 6.**
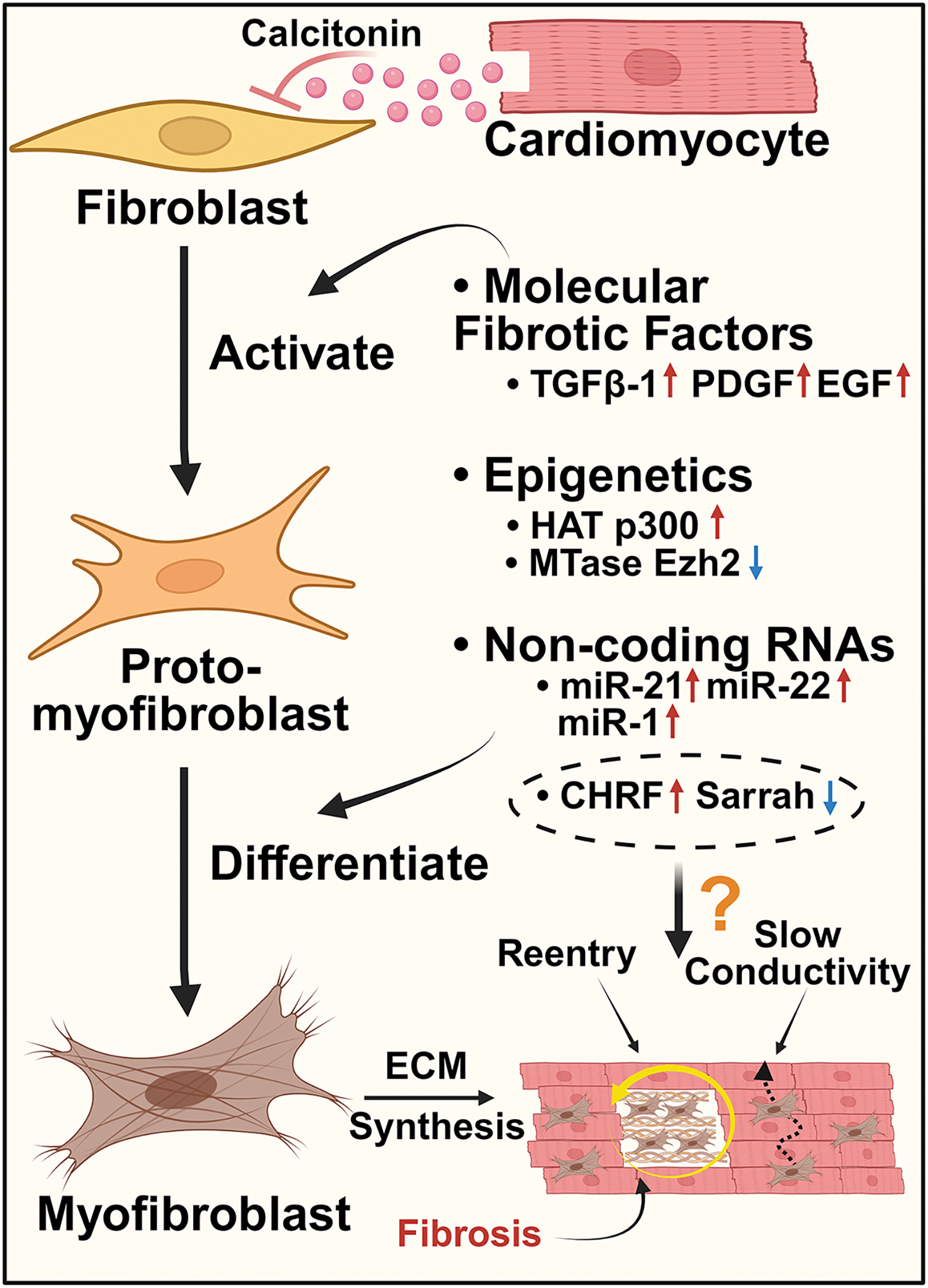
Fibrosis in aging and AF. Fibrosis is an age-associated change in the heart. Atrial fibrosis is characterized by increased proliferation and activation of fibroblasts into intermediate proto-myofibroblasts and subsequently mature myofibroblast phenotypes. This process is regulated by molecular fibrotic factors such as TGFβ-1, PDGF, and EGF, epigenetic mechanisms, and non-coding RNAs. Histone acetyltransferase (HAT) p300 is upregulated with aging in AF, while methyltransferase (MTase) Ezh2 is downregulated in aged AF mice. Among non-coding RNAs, microRNAs including miR-21, miR-22, and miR-1 are increased in aging, correlating with the degree of myocardial fibrosis and AF progression. Alterations in long non-coding RNAs such as *CHRF* and *Sarrah* have been observed in aging, albeit their direct association with AF occurrence requires further study (indicated by a question mark). Paracrine signaling, such as calcitonin from cardiomyocytes, exerts an inhibitory effect on fibroblast activation. However, calcitonin produced by atrial cardiomyocytes (ACMs) decreases with aging. Myofibroblast contributes to excessive collagen production in the atrial myocardium, leading to electrical/structural changes such as increased atrial stiffness, slow conductivity, formation of reentry circuits, and increased risk of ectopic activity, promoting and sustaining AF. ECM: Extracellular matrix; TGFβ-1: transforming growth factor beta 1; PDGF: platelet-derived growth factor; EGF: epidermal growth factor. The figure was generated with BioRender.com.

The aging heart exhibits enhanced myocardial fibrosis. Fibrosis, viewed as the terminal stage of chronic inflammation, is established by the excessive deposition and post-translational modification of collagen, fibronectin, and other extracellular matrix (ECM) proteins in the atrial myocardium by activated, profibrotic cardiac fibroblasts. These age-dependent changes in ECM composition characterized by enhanced collagen deposition contribute to increased tissue stiffness and diastolic dysfunction^[134]^. Age-dependent neurohormonal factors promote increased cardiac fibroblast proliferation and their transition to an intermediate proto-myofibroblast and finally to a myofibroblast state, characterized by alpha-smooth muscle actin (αSMA) expression [Figure 6]^[135]^. In the aging heart, growth factor signaling and cardiac fibroblast are perturbed to favor enhanced ECM deposition and proinflammatory fibroblast phenotypes^[136]^. Mechanistically, profibrotic age-dependent changes include downregulated canonical TGF-β1 signaling [Figure 6]^[137]^. Moreover, there is evidence for increased ERK activation by non-canonical TGF-β1 signaling as well as insulin, PDGF, EGF, and angiotensin II, which promote collagen production [Figure 6]^[137–139]^. Excessive fibrosis, in turn, increases conduction heterogeneity and enhances myocardial stiffness which can progress to diastolic dysfunction^[136]^.

### Cardiac fibrosis establishes a permissive AF substrate

Cardiac fibrosis, a hallmark of structural remodeling, promotes pathological changes predominantly in the atria in AF, providing a substrate for AF by increasing conduction heterogeneity and stabilizing reentrant circuits. Fibrosis in AF has been confirmed by cardiac magnetic resonance (CMR) imaging and electroanatomical mapping, as well as histological studies of the myocardium in human and mouse hearts^[140,141]^. Moreover, enhanced left atrial fibrosis in mAF is associated with worse cardiovascular outcomes^[142]^. The CREM-IbΔC-X transgenic (CREM-Tg) mice, which exhibit significant age-dependent progressive atrial remodeling and spontaneous AF, provide a useful experimental model to study the role of fibrosis in AF. Notably, the onset of spontaneous AF temporally coincides with the development of significant atrial fibrosis^[143,144]^. Unbiased proteomics analyses by our team discovered a distinct enrichment of fibrotic processes in chronic AF in CREM-Tg mice at 9 months of age, whereas in younger mice (7 weeks), the most enriched biological processes were primarily related to ion channel and metabolic dysregulation in young CREM-Tg mice^[145]^. These findings reveal the critical involvement of fibrosis in AF progression, emphasizing the shift in the pathogenic contribution from electrical to structural cardiac remodeling during the aging process.

Fibrotic remodeling increases ECM stiffness, which further triggers fibroblast activation, creating a positive feedback loop^[146]^. In addition, activated fibroblasts in the aging heart release selective inflammatory mediators (e.g., IL-6), indicating a shift toward a proinflammatory phenotype that enhances leukocyte recruitment^[147]^. Notably, single nuclei RNA sequencing of 12-week-old *vs.* 18-month-old mice identified aged fibroblasts as the cell type with the most significant differential gene expression pattern including an enhanced inflammatory secretion profile^[148]^. Together, these alterations in cardiac fibroblast activity impair heart function and elevate the risk of arrhythmias. A clinical study in older AF patients (> 60 years old) showed that cardiac fibroblasts developed a premature senescence phenotype, which is accompanied by increased fibrosis in persistent AF patients, pointing toward the possibility of targeting premature senescence as an antifibrotic therapy in age-related AF^[149]^.

Paracrine signaling plays an important role in fibroblast activation and can promote dysfunction of neighboring cell types. In the heart, intercellular communication between cardiomyocytes and cardiac fibroblasts is mediated by paracrine signaling^[150]^. Cardiotrophin-1, a cytokine produced by cardiomyocytes, stimulates hypertrophic responses in cardiomyocytes and promotes fibroblast proliferation^[151]^. Although cardiotrophin-1 deletion in mice suppressed age-dependent vascular remodeling^[152]^, its role in the remodeling of the aging myocardium remains to be investigated. Calcitonin, a hormone produced by the thyroid that negatively regulates systemic Ca^2+^ homeostasis, has been shown to be secreted in substantial amounts by atrial cardiomyocytes and exert cardioprotective effects^[153]^. Cardiac calcitonin activates calcitonin receptors on neighboring atrial cardiac fibroblasts to regulate their proliferation, migratory activity, and secretion of ECM proteins [Figure 6]^[153]^. Compared to individuals in sinus rhythm, myocardial production of calcitonin was significantly reduced in patients with persistent AF. Additionally, a negative correlation was found between myocardial calcitonin protein levels and age. These observations suggest that age-dependent depletion of myocardial calcitonin may promote paracrine-mediated fibrogenesis in the atria and perpetuate AF^[153]^. Future research should aim to elucidate strategies to restore aberrant calcitonin-calcitonin receptor signaling in AF atrial fibroblasts to reduce and/or reverse the structural fibrosis.

### Epigenetic regulation of cardiac fibrosis

A recent work conducted by Gao *et al.* provided an epigenetic perspective to targeting senescence in atrial cardiac fibroblasts^[154]^. Acetyltransferase p300, an epigenetic regulator, has been recognized for its roles in cellular senescence, fibrosis, and other age-related processes^[155]^. In older AF patients (> 50 years old) and aged mice (> 18 months old), the expression level of p300 was significantly increased [Figure 6]. Both pharmacological and genetic inhibition of p300 reduced atrial fibroblast senescence and atrial fibrosis, resulting in decreased AF inducibility. In contrast, overexpression of p300 led to enhanced senescence of atrial fibroblasts and atrial fibrosis. The contribution of p300 to senescence and atrial fibrosis was found to be mediated through the p53/Smad3 pathway^[154]^. In a similar vein, Li *et al.* explored epigenetic regulation in age-related AF from a histone methylation perspective^[156]^. Their study showed decreased production of methyltransferase Ezh2 in the atria of aged mice with AF [Figure 6] and unveiled that Ezh2 restored H3K27me3 levels in the promoter regions of *CDKN2a* and *Timp4* genes in primary mouse atrial fibroblasts at late passages, preventing senescence development and alleviating profibrotic effects^[156]^. Together, these studies indicate that future interventions at the epigenetic level could potentially modulate histone acetylation and methylation patterns to prevent the development of an AF substrate. Such modulations could regulate gene expression related to atrial fibroblast function and senescence, offering a new approach for mitigating age-related processes that contribute to AF onset and progression.

### Non-coding RNAs and atrial fibrogenesis

Another field garnering attention in the investigation of mechanisms driving atrial fibrosis in AF is the regulation of non-coding RNAs, including microRNA (miRNA) and long non-coding RNA (lncRNA). Accumulating evidence has established altered miRNA expression patterns as biomarkers and regulators of longevity and tissue-specific aging processes^[157]^. Elucidating the relationship between miRNA and atrial fibrosis could provide novel insights into the initiation and maintenance of age-associated AF. miR-21 was the first miRNA reported to contribute to atrial fibrogenesis^[158]^. In the left atrial myocardium of elderly AF patients, miR-21 production is increased [Figure 6] and accompanied by downregulation of its target Spry1. Pharmacological inhibition of miR-21 *in vivo* led to a reduction in interstitial atrial fibrosis^[158]^. Moreover, elevated levels of another miRNA, miR-22, in the hearts of aging mice have a role in promoting cardiac fibroblast senescence [Figure 6]^[159]^. In a tachypacing-induced canine AF model, miR-26 was found to be negatively regulated by the nuclear factor of activated T cells (NFAT), and the downregulation of miR-26 led to altered atrial cardiac fibroblast proliferation, potentially promoting fibrotic remodeling in the atria^[160]^. Interestingly, NFAT inhibition was later reported to protect against atrial fibrosis and AF development in CREM-Tg model with spontaneous AF (at 7 months of age)^[143]^. Recently, the expression of miR-1 was found to be highly elevated in elderly AF patients (> 65 years old) and highly correlated with the degree of myocardial fibrosis [Figure 6], although the detailed mechanisms underpinning the correlation remain unknown^[161]^. Thus, the pervasive atrial fibrosis in persistent AF might be attributed to altered modulation of miRNA expression during aging. In addition to contributing to the structural substrate, dysregulated miRNA contributes to electrical remodeling; it was shown that miR-31 is upregulated in AF and depletes neuronal NOS and dystrophin, leading to shortened APD^[162]^. Meanwhile, miR-31 is also time-dependently upregulated following AMI in rats and its inhibition ameliorates maladaptive post-AMI cardiac remodeling^[163]^. Therefore, miR-31 may play a role in the development of the AF substrate prior to AF occurrence, particularly in those with previous AMI or heart failure.

Compared to miRNAs, the understanding of long non-coding RNAs (lncRNAs) in the aging heart is limited, although a few lncRNA targets have been associated with other cardiovascular diseases [Figure 6]. For example, *MALAT1* enhanced profibrotic responses in a myocardial infarction mouse model through the regulation of TGF-β1 signaling^[164]^. Alongside fibrosis, cardiac hypertrophy is developed during aging due to elevated levels of angiotensin II^[165]^. *CHRF* is a lncRNA that could potentially promote hypertrophy in the aging heart. It suppresses the activities of *miR-93* and *miR-489*, resulting in Akt3-induced and Myd88-induced hypertrophy, respectively^[166,167]^. *Sarrah* serves as an anti-apoptotic lncRNA in cardiomyocytes after acute myocardial infarction (AMI) and was demonstrated to be downregulated in aging^[168]^. Other lncRNAs, including *MIRT1* and *MIRT2*, are upregulated after AMI, and promote left ventricular remodeling^[169]^. Although these lncRNAs were explored in the context of other cardiac diseases, the underlying changes they cause are closely related to those involved in AF pathogenesis. Furthermore, these cardiovascular diseases are commonly identified as comorbidities during aging.

While RNA-based drugs have shown promise and have been approved for clinical use, improving the efficiency of RNA drug delivery remains challenging due to issues with stability and off-target effects, potentially limiting their full translational potential^[170,171]^. Future research on RNA-based therapeutics in atrial fibrosis and AF should focus not only on the regulation of miRNAs or lncRNAs, but also on upstream factors (transcriptional factors or RNA binding proteins) and downstream targets. This will provide a more comprehensive understanding of how non-coding RNAs modulate fibrosis and AF, offering a novel approach to treating age-associated AF. The relationship between aging and AF in fibrosis is summarized in Figure 6.

## LOSS OF PROTEOSTASIS

**Figure 7.**
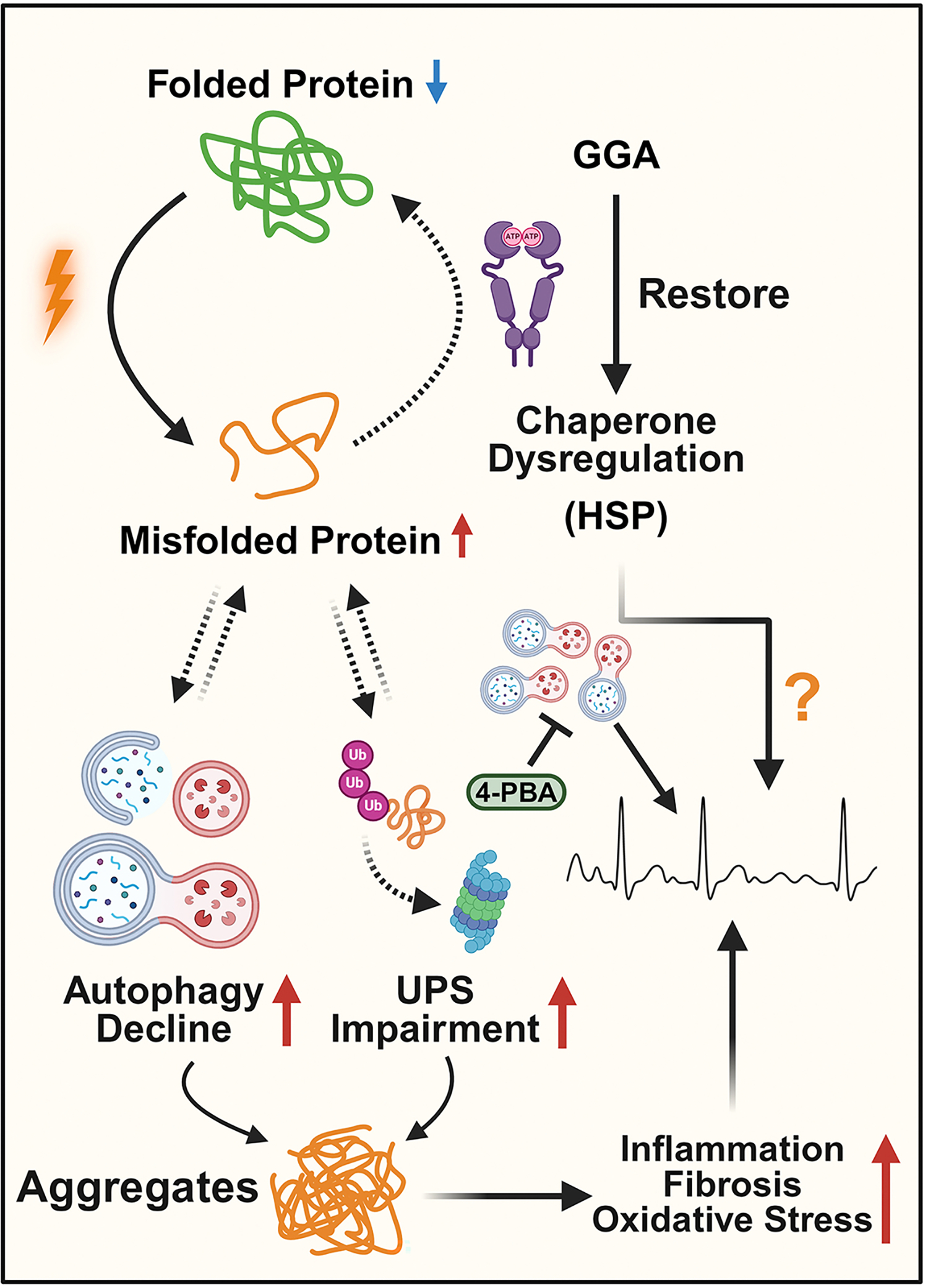
Loss of proteostasis in aging and AF. An impairment of proteostasis during aging underlies dysregulation of autophagy and the ubiquitination-proteasome system (UPS), leading to the accumulation of misfolded protein aggregates. These aggregates can be managed by restoring the function of molecular chaperones such as heat shock protein (HSP) by geranylgeranylacetone (GGA). Whether this restoration of HSP in the aging heart could prevent AF remains uncertain (indicated by a question mark). Notably, AF may be associated with excessive autophagy activation due to ER stress, which can be inhibited by 4-Phenylbutyric acid (4-PBA) treatment. The unresolved aggregates induce cellular stress, contributing to inflammation and fibrosis in the heart, eventually creating an arrhythmogenic substrate that allows for the occurrence and perpetuation of AF. Dashed lines indicate disruptions in normal physiological processes, leading to age-related consequences. The figure was generated with BioRender.com.

Protein homeostasis, also termed proteostasis, collectively refers to the myriad of cellular mechanisms that ensure the proteome’s stability and proper function by ensuring appropriate protein synthesis, post-translational modification, transport, recycling, and degradation. AF, alongside many other age-related maladies, has been associated with a failure to maintain proper proteostasis^[4,5]^. Proteostasis is conferred largely by the protein quality control (PQC) system, which comprises protein chaperones, to assist with proper protein folding, and the autophagy and ubiquitin-proteasome molecular machinery to clear aberrantly folded or aggregated proteins [Figure 7]. Cardiomyocytes are formidable protein synthesis factories, producing around 14,000 proteins, rendering them particularly vulnerable to accumulating toxic levels of misfolded proteins in times of stress, such as in AF due to the rapid activation rates and enhanced mechanical and metabolic stress^[8]^. In response to cellular stress, the heat shock response upregulates the expression of heat shock proteins, a group of protein chaperones. In AF, levels of heat shock protein 27 (HSP27) are initially upregulated in paroxysmal AF, localizing to the contractile apparatus, but are gradually exhausted as AF progresses^[172]^. Furthermore, the pharmacological induction of HSP expression with geranylgeranylacetone (GGA) is antiarrhythmic in both *in vitro* HL-1 cells and *in vivo* dogs, and oral GGA treatment has been shown to induce atrial HSP expression in humans undergoing cardiac surgery [Figure 7]^[173,174]^. Whether therapeutic activation of the HSR in the aging heart could prevent the initiation or progression of AF has not been explored.

Concomitant with dysregulated protein folding in aging and AF is the decline in the capacity of proteolytic systems to clear misfolded or aggregated proteins and damaged organelles. Autophagy is a highly conserved process that clears damaged proteins and organelles by packaging them into autophagosomes, which subsequently fuse with lysosomes, leading to the degradation of the autophagosome contents. Pathological aging processes are generally linked with a decline in autophagic capacity [Figure 7]. In a recent clinical study of 150 AF patients and 150 age- and sex-matched controls, compromised autophagy was observed in AF patients, particularly in older individuals^[175]^. On the other hand, there are examples of age-related pathologies, like AF, that are associated with excessive autophagy. In experimental models of AF and in atrial appendage samples from patients with AF, cardiomyocyte autophagy was found to be activated by enhanced ER stress, which could be attenuated by treatment with the chemical chaperone 4-PBA [Figure 7]^[176]^. Excessive activation of autophagy is associated with myolysis and thus emerges as a molecular mechanism of AF-associated cardiac remodeling. Similarly, as cytosolic Ca^2+^ levels increase, there is activation of calpain 1, a Ca^2+^-dependent neutral cysteine protease, in both paroxysmal and persistent AF, which, alongside autophagy, further enhances myolysis^[177]^. Enhanced calpain activity has also been described as an important phenomenon in cellular aging and has been implicated in many age-related pathologies^[178]^. These findings suggest the importance of identifying specific targets within the proteolytic pathways to help maintain degradative activities at optimal levels rather than bluntly activating or inhibiting them, to potentially mitigate cardiovascular risk in AF. The relationship between aging and AF in impaired proteostasis is summarized in Figure 7.

## CONCLUSIONS AND FUTURE PERSPECTIVE

Aging remains the most strongly associated risk factor for AF, yet a precise delineation of the molecular and cellular changes that underpin this association is not available^[1]^. Decades of collaborative efforts have identified key alterations that occur with aging, which may promote the electrophysiological and anatomical changes in the heart, making it vulnerable to AF. The mechanisms highlighted in this review do not occur in isolation, but instead are highly interconnected and often mutually reinforcing. For instance, oxidative stress may lead to, and be caused by, mitochondrial dysfunction which leads to the release of mitochondrial DNA and other mitochondrial components into the cytosol, triggering cell senescence, thereby enhancing inflammation via activation of the SASP, which, in turn, may promote localized tissue fibrosis.

The accumulation of age-related damage, determined by a patient’s exposome and comorbidities, layered on their individual genetic predisposition, likely distinguishes those who will develop AF as they age *vs.* those who will not. Charting the development timeline of these age-hallmarking pathological processes might pave the way toward more tailored and effective therapeutics that specifically target the most essential process, at the most appropriate time and in the most suitable patients. It is also noteworthy that women live longer and have a lower risk for AF, typically developing it at an older age and with unique prevailing mechanisms, compared to men^[179,180]^. The possibility of shared genetic and hormonal mechanisms underpinning the observed sex differences in mortality and AF risk requires further scrutiny with detailed mechanistic investigations. New insights may yield sex-tailored therapies that improve the outcomes of current age-related AF treatment options^[180]^. Although increasing chronological age is an immutable risk factor, recent findings have found that AF incidence is also independently associated with epigenetic markers of biological aging, which can be delayed or even reversed by lifestyle intervention or treatment strategies^[181,182]^. The identification of emerging mechanisms (e.g., microbiome dysbiosis or metabolite deficiencies) that can be addressed with low-cost dietary and broader lifestyle interventions is a particularly promising avenue for the management of this increasingly prevalent arrhythmia. To accelerate clinical translation, future research should aim to:

- Unravel which hallmarks of aging are merely consequences or passive biomarkers of AF *vs.* those that casually contribute to the disease pathogenesis.
- Develop, characterize and standardize more relevant animal models of spontaneous, age-related AF.
- Standardize preclinical and clinical definitions of advanced age in the context of age-related AF to help synchronize the interpretation of results.
- Harness and advance recent improvements in patient-specific iPSC platforms to generate precise and effective drug screening assays that overcome the limitations of interspecies differences.

With new insights into the mechanisms that causally couple advancing age to AF, it is hoped that tomorrow’s clinician will be armed with an arsenal of novel, holistic lifestyle and pharmacological interventions that can sever the link between the non-modifiable process of chronological aging and AF.

## Data Availability

Original data sets are available from the corresponding author upon reasonable request.

## References

[1] Elliott AD, Middeldorp ME, Van Gelder IC, Albert CM, Sanders P. Epidemiology and modifiable risk factors for atrial fibrillation. Nat Rev Cardiol 2023;20:404–17. 36600003 10.1038/s41569-022-00820-8

[2] Chen Q, Yi Z, Cheng J. Atrial fibrillation in aging population. Aging Med 2018;1:67–74. 10.1002/agm2.12015 PMC6880740 31942483

[3] Chen C, Ding S, Wang J. Digital health for aging populations. Nat Med 2023;29:1623–30. 37464029 10.1038/s41591-023-02391-8

[4] López-Otín C, Blasco MA, Partridge L, Serrano M, Kroemer G. Hallmarks of aging: an expanding universe. Cell 2023;186:243–78. 36599349 10.1016/j.cell.2022.11.001

[5] López-Otín C, Blasco MA, Partridge L, Serrano M, Kroemer G. The hallmarks of aging. Cell 2013;153:1194–217. 23746838 10.1016/j.cell.2013.05.039 PMC3836174

[6] Partridge L, Fuentealba M, Kennedy BK. The quest to slow ageing through drug discovery. Nat Rev Drug Discov 2020;19:513–32. 32467649 10.1038/s41573-020-0067-7

[7] Haïssaguerre M, Jaïs P, Shah DC, Spontaneous initiation of atrial fibrillation by ectopic beats originating in the pulmonary veins. N Engl J Med 1998;339:659–66. 9725923 10.1056/NEJM199809033391003

[8] Brundel BJJM, Ai X, Hills MT, Kuipers MF, Lip GYH, de Groot NMS. Atrial fibrillation. Nat Rev Dis Primers 2022;8:21. 35393446 10.1038/s41572-022-00347-9

[9] Wijffels MCEF, Kirchhof CJHJ, Dorland R, Allessie MA. Atrial fibrillation begets atrial fibrillation: a study in awake chronically instrumented goats. Circulation 1995;92:1954–68. 7671380 10.1161/01.cir.92.7.1954

[10] Camm AJ, Naccarelli GV, Mittal S, The increasing role of rhythm control in patients with atrial fibrillation: JACC state-of-the-art review. J Am Coll Cardiol 2022;79:1932–48. 35550691 10.1016/j.jacc.2022.03.337

[11] Dye CA, Skeete J, Khan A, The era of rhythm control: a review of the epidemiology and clinical impact of anti-arrhythmic medications in atrial fibrillation. Pharmacoepidemiology 2023;2:81–97.

[12] Tisdale JE, Chung MK, Campbell KB, Drug-induced arrhythmias: a scientific statement from the American heart association. Circulation 2020;142:e214–33. 32929996 10.1161/CIR.0000000000000905

[13] Hindricks G, Potpara T, Dagres N, 2020 ESC Guidelines for the diagnosis and management of atrial fibrillation developed in collaboration with the European Association for Cardio-Thoracic Surgery (EACTS): The Task Force for the diagnosis and management of atrial fibrillation of the European Society of Cardiology (ESC) Developed with the special contribution of the European Heart Rhythm Association (EHRA) of the ESC. Eur Heart J 2021;42:373–498. 32860505 10.1093/eurheartj/ehaa612

[14] Brooks S, Metzner A, Wohlmuth P, Insights into ablation of persistent atrial fibrillation: Lessons from 6-year clinical outcomes. J Cardiovasc Electrophysiol 2018;29:257–63. 29216412 10.1111/jce.13401

[15] Jaakkola S, Lip GY, Biancari F, Predicting unsuccessful electrical cardioversion for acute atrial fibrillation (from the AF-CVS Score). Am J Cardiol 2017;119:749–52. 28017305 10.1016/j.amjcard.2016.11.026

[16] Lahiri SK, Aguilar-Sanchez Y, Wehrens XHT. Mechanisms underlying pathological Ca^2+^ handling in diseases of the heart. Pflugers Arch 2021;473:331–47. 33399957 10.1007/s00424-020-02504-z PMC10070045

[17] Biliczki P, Boon RA, Girmatsion Z, Age-related regulation and region-specific distribution of ion channel subunits promoting atrial fibrillation in human left and right atria. Europace 2019;21:1261–9. 31131392 10.1093/europace/euz135

[18] Heijman J, Voigt N, Nattel S, Dobrev D. Cellular and molecular electrophysiology of atrial fibrillation initiation, maintenance, and progression. Circ Res 2014;114:1483–99. 24763466 10.1161/CIRCRESAHA.114.302226

[19] Turdi S, Fan X, Li J, AMP-activated protein kinase deficiency exacerbates aging-induced myocardial contractile dysfunction. Aging Cell 2010;9:592–606. 20477759 10.1111/j.1474-9726.2010.00586.x PMC2910211

[20] Qin F, Siwik DA, Lancel S, Hydrogen peroxide-mediated SERCA cysteine 674 oxidation contributes to impaired cardiac myocyte relaxation in senescent mouse heart. J Am Heart Assoc 2013;2:e000184. 23963753 10.1161/JAHA.113.000184 PMC3828801

[21] Guo KK, Ren J. Cardiac overexpression of alcohol dehydrogenase (ADH) alleviates aging-associated cardiomyocyte contractile dysfunction: role of intracellular Ca2+ cycling proteins. Aging Cell 2006;5:259–65. 16842498 10.1111/j.1474-9726.2006.00215.x

[22] Gergs U, Mangold W, Langguth F, Alterations of protein expression of phospholamban, ZASP and plakoglobin in human atria in subgroups of seniors. Sci Rep 2019;9:5610. 30948763 10.1038/s41598-019-42141-w PMC6449388

[23] Salameh A, Dhein S, Fleischmann B, The aging heart: changes in the pharmacodynamic electrophysiological response to verapamil in aged rabbit hearts. J Physiol Pharmacol 2010;61:141–51. 20436214

[24] Isenberg G, Borschke B, Rueckschloss U. Ca^2+^ transients of cardiomyocytes from senescent mice peak late and decay slowly. Cell Calcium 2003;34:271–80. 12887974 10.1016/s0143-4160(03)00121-0

[25] Valdés Á, Treuer AV, Barrios G, NOX inhibition improves β-adrenergic stimulated contractility and intracellular calcium handling in the aged rat heart. Int J Mol Sci 2018;19:2404. 30111689 10.3390/ijms19082404 PMC6121436

[26] Zhu X, Altschafl BA, Hajjar RJ, Valdivia HH, Schmidt U. Altered Ca^2+^ sparks and gating properties of ryanodine receptors in aging cardiomyocytes. Cell Calcium 2005;37:583–91. 15862349 10.1016/j.ceca.2005.03.002

[27] Wongcharoen W, Chen YC, Chen YJ, Aging increases pulmonary veins arrhythmogenesis and susceptibility to calcium regulation agents. Heart Rhythm 2007;4:1338–49. 17905341 10.1016/j.hrthm.2007.06.023

[28] Cooper LL, Li W, Lu Y, Redox modification of ryanodine receptors by mitochondria-derived reactive oxygen species contributes to aberrant Ca^2+^ handling in ageing rabbit hearts. J Physiol 2013;591:5895–911. 24042501 10.1113/jphysiol.2013.260521 PMC3872760

[29] Marx SO, Marks AR. Dysfunctional ryanodine receptors in the heart: new insights into complex cardiovascular diseases. J Mol Cell Cardiol 2013;58:225–31. 23507255 10.1016/j.yjmcc.2013.03.005 PMC4042628

[30] Wehrens XH, Lehnart SE, Reiken S, Vest JA, Wronska A, Marks AR. Ryanodine receptor/calcium release channel PKA phosphorylation: a critical mediator of heart failure progression. Proc Natl Acad Sci USA 2006;103:511–8. 16407108 10.1073/pnas.0510113103 PMC1334677

[31] Uchinoumi H, Yang Y, Oda T, CaMKII-dependent phosphorylation of RyR2 promotes targetable pathological RyR2 conformational shift. J Mol Cell Cardiol 2016;98:62–72. 27318036 10.1016/j.yjmcc.2016.06.007 PMC5026590

[32] Wehrens XH, Lehnart SE, Reiken SR, Marks AR. Ca^2+^/calmodulin-dependent protein kinase II phosphorylation regulates the cardiac ryanodine receptor. Circ Res 2004;94:e61–70. 15016728 10.1161/01.RES.0000125626.33738.E2

[33] Campbell HM, Quick AP, Abu-Taha I, Loss of SPEG inhibitory phosphorylation of ryanodine receptor type-2 promotes atrial fibrillation. Circulation 2020;142:1159–72. 32683896 10.1161/CIRCULATIONAHA.120.045791 PMC7508800

[34] Mendonca N, Ling S, Bedja D, Dysregulation of cardiac CaMKII pathway is increased in aging and chronic inflammation. FASEB J 2021;35.

[35] Beavers DL, Landstrom AP, Chiang DY, Wehrens XH. Emerging roles of junctophilin-2 in the heart and implications for cardiac diseases. Cardiovasc Res 2014;103:198–205. 24935431 10.1093/cvr/cvu151 PMC4809974

[36] Beavers DL, Wang W, Ather S, Mutation E169K in junctophilin-2 causes atrial fibrillation due to impaired RyR2 stabilization. J Am Coll Cardiol 2013;62:2010–9. 23973696 10.1016/j.jacc.2013.06.052 PMC3830688

[37] Munro ML, Jayasinghe I, Wang Q, Junctophilin-2 in the nanoscale organisation and functional signalling of ryanodine receptor clusters in cardiomyocytes. J Cell Sci 2016;129:4388–98. 27802169 10.1242/jcs.196873 PMC5201013

[38] Reynolds JO, Quick AP, Wang Q, Junctophilin-2 gene therapy rescues heart failure by normalizing RyR2-mediated Ca2+ release. Int J Cardiol 2016;225:371–80. 27760414 10.1016/j.ijcard.2016.10.021 PMC5101129

[39] Lyu Y, Verma VK, Lee Y, Remodeling of t-system and proteins underlying excitation-contraction coupling in aging versus failing human heart. NPJ Aging Mech Dis 2021;7:16. 34050186 10.1038/s41514-021-00066-7 PMC8163749

[40] Choi S, Vivas O, Baudot M, Moreno CM. Aging alters the formation and functionality of signaling microdomains between L-type calcium channels and β2-adrenergic receptors in cardiac pacemaker cells. Front Physiol 2022;13:805909. 35514336 10.3389/fphys.2022.805909 PMC9065441

[41] Luo X, Yu W, Liu Z, Ageing increases cardiac electrical remodelling in rats and mice via NOX4/ROS/CaMKII-mediated calcium signalling. Oxid Med Cell Longev 2022;2022:8538296. 35387264 10.1155/2022/8538296 PMC8979732

[42] Anyukhovsky EP, Sosunov EA, Plotnikov A, Cellular electrophysiologic properties of old canine atria provide a substrate for arrhythmogenesis. Cardiovasc Res 2002;54:462–9. 12062351 10.1016/s0008-6363(02)00271-7

[43] Herraiz-Martínez A, Álvarez-García J, Llach A, Ageing is associated with deterioration of calcium homeostasis in isolated human right atrial myocytes. Cardiovasc Res 2015;106:76–86. 25712961 10.1093/cvr/cvv046 PMC4362404

[44] Feridooni HA, Dibb KM, Howlett SE. How cardiomyocyte excitation, calcium release and contraction become altered with age. J Mol Cell Cardiol 2015;83:62–72. 25498213 10.1016/j.yjmcc.2014.12.004

[45] Joseph LC, Reyes MV, Homan EA, The mitochondrial calcium uniporter promotes arrhythmias caused by high-fat diet. Sci Rep 2021;11:17808. 34497331 10.1038/s41598-021-97449-3 PMC8426388

[46] Liu C, Bai J, Dan Q, Mitochondrial dysfunction contributes to aging-related atrial fibrillation. Oxid Med Cell Longev 2021;2021:5530293. 34007402 10.1155/2021/5530293 PMC8102104

[47] Liguori I, Russo G, Curcio F, Oxidative stress, aging, and diseases. Clin Interv Aging 2018;13:757–72. 29731617 10.2147/CIA.S158513 PMC5927356

[48] Harman D Aging: a theory based on free radical and radiation chemistry. J Gerontol 1956;11:298–300. 13332224 10.1093/geronj/11.3.298

[49] Martínez de Toda I, Ceprián N, Díaz-Del Cerro E, De la Fuente M. The role of immune cells in oxi-inflamm-aging. Cells 2021;10:2974. 34831197 10.3390/cells10112974 PMC8616159

[50] Gladyshev VN. The free radical theory of aging is dead. Long live the damage theory! Antioxid Redox Signal 2014;20:727–31. 24159899 10.1089/ars.2013.5228 PMC3901353

[51] Tan BL, Norhaizan ME, Liew WP, Sulaiman Rahman H. Antioxidant and oxidative stress: a mutual interplay in age-related diseases. Front Pharmacol 2018;9:1162. 30405405 10.3389/fphar.2018.01162 PMC6204759

[52] Maldonado E, Morales-Pison S, Urbina F, Solari A. Aging hallmarks and the role of oxidative stress. Antioxidants 2023;12:651. 36978899 10.3390/antiox12030651 PMC10044767

[53] Iakovou E, Kourti M. A comprehensive overview of the complex role of oxidative stress in aging, the contributing environmental stressors and emerging antioxidant therapeutic interventions. Front Aging Neurosci 2022;14:827900. 35769600 10.3389/fnagi.2022.827900 PMC9234325

[54] Peoples JN, Saraf A, Ghazal N, Pham TT, Kwong JQ. Mitochondrial dysfunction and oxidative stress in heart disease. Exp Mol Med 2019;51:1–13. 10.1038/s12276-019-0355-7 PMC6923355 31857574

[55] Reilly SN, Jayaram R, Nahar K, Atrial sources of reactive oxygen species vary with the duration and substrate of atrial fibrillation: implications for the antiarrhythmic effect of statins. Circulation 2011;124:1107–17. 21844076 10.1161/CIRCULATIONAHA.111.029223

[56] Rudolph V, Andrié RP, Rudolph TK, Myeloperoxidase acts as a profibrotic mediator of atrial fibrillation. Nat Med 2010;16:470–4. 20305660 10.1038/nm.2124 PMC2880896

[57] Youn JY, Zhang J, Zhang Y, Oxidative stress in atrial fibrillation: an emerging role of NADPH oxidase. J Mol Cell Cardiol 2013;62:72–9. 23643589 10.1016/j.yjmcc.2013.04.019 PMC3735724

[58] Xie W, Santulli G, Reiken SR, Mitochondrial oxidative stress promotes atrial fibrillation. Sci Rep 2015;5:11427. 26169582 10.1038/srep11427 PMC4501003

[59] D’Oria R, Schipani R, Leonardini A, The role of oxidative stress in cardiac disease: from physiological response to injury factor. Oxid Med Cell Longev 2020;2020:5732956. 32509147 10.1155/2020/5732956 PMC7244977

[60] Liu H, Wang Q, Liu D, Manganese superoxide dismutase as a novel oxidative stress biomarker for predicting paroxysmal atrial fibrillation. J Clin Med 2022;11:5131. 36079059 10.3390/jcm11175131 PMC9457192

[61] Kim YH, Lim DS, Lee JH, Gene expression profiling of oxidative stress on atrial fibrillation in humans. Exp Mol Med 2003;35:336–49. 14646586 10.1038/emm.2003.45

[62] Kozakiewicz M, Kornatowski M, Krzywińska O, Kędziora-Kornatowska K. Changes in the blood antioxidant defense of advanced age people. Clin Interv Aging 2019;14:763–71. 31118597 10.2147/CIA.S201250 PMC6507109

[63] Watts M, Kolluru GK, Dherange P, Decreased bioavailability of hydrogen sulfide links vascular endothelium and atrial remodeling in atrial fibrillation. Redox Biol 2021;38:101817. 33310503 10.1016/j.redox.2020.101817 PMC7732878

[64] Tesauro M, Mauriello A, Rovella V, Arterial ageing: from endothelial dysfunction to vascular calcification. J Intern Med 2017;281:471–82. 28345303 10.1111/joim.12605

[65] Chong E, Chang SL, Hsiao YW, Resveratrol, a red wine antioxidant, reduces atrial fibrillation susceptibility in the failing heart by PI3K/AKT/eNOS signaling pathway activation. Heart Rhythm 2015;12:1046–56. 25640634 10.1016/j.hrthm.2015.01.044

[66] Dong Q, Wu Z, Li X, Resveratrol ameliorates cardiac dysfunction induced by pressure overload in rats via structural protection and modulation of Ca^2+^ cycling proteins. J Transl Med 2014;12:323. 25425099 10.1186/s12967-014-0323-x PMC4278670

[67] Csengeri D, Sprünker NA, Di Castelnuovo A, Alcohol consumption, cardiac biomarkers, and risk of atrial fibrillation and adverse outcomes. Eur Heart J 2021;42:1170–7. 33438022 10.1093/eurheartj/ehaa953 PMC7982286

[68] Baczkó I, Light PE. Resveratrol and derivatives for the treatment of atrial fibrillation. Ann N Y Acad Sci 2015;1348:68–74. 26205342 10.1111/nyas.12843

[69] Wang C, Pan Z. Hydrogen-rich saline mitigates pressure overload-induced cardiac hypertrophy and atrial fibrillation in rats via the JAK-STAT signalling pathway. J Int Med Res 2020;48:300060520936415. 32762484 10.1177/0300060520936415 PMC7416141

[70] Mighiu AS, Recalde A, Ziberna K, Inducibility, but not stability, of atrial fibrillation is increased by NOX2 overexpression in mice. Cardiovasc Res 2021;117:2354–64. 33483749 10.1093/cvr/cvab019 PMC8479801

[71] Kim YM, Guzik TJ, Zhang YH, A myocardial Nox2 containing NAD(P)H oxidase contributes to oxidative stress in human atrial fibrillation. Circ Res 2005;97:629–36. 16123335 10.1161/01.RES.0000183735.09871.61

[72] Patti G, Chello M, Candura D, Randomized trial of atorvastatin for reduction of postoperative atrial fibrillation in patients undergoing cardiac surgery: results of the ARMYDA-3 (Atorvastatin for Reduction of MYocardial Dysrhythmia After cardiac surgery) study. Circulation 2006;114:1455–61. 17000910 10.1161/CIRCULATIONAHA.106.621763

[73] Shiroshita-Takeshita A, Schram G, Lavoie J, Nattel S. Effect of simvastatin and antioxidant vitamins on atrial fibrillation promotion by atrial-tachycardia remodeling in dogs. Circulation 2004;110:2313–9. 15477401 10.1161/01.CIR.0000145163.56529.D1

[74] Zheng Z, Jayaram R, Jiang L, Perioperative rosuvastatin in cardiac surgery. N Engl J Med 2016;374:1744–53. 27144849 10.1056/NEJMoa1507750

[75] Murphy C, Deplazes E, Cranfield CG, Garcia A. The role of structure and biophysical properties in the pleiotropic effects of statins. Int J Mol Sci 2020;21:8745. 33228116 10.3390/ijms21228745 PMC7699354

[76] Rahimi K, Emberson J, McGale P, Effect of statins on atrial fibrillation: collaborative meta-analysis of published and unpublished evidence from randomised controlled trials. BMJ 2011;342:d1250. 21411487 10.1136/bmj.d1250

[77] El Assar M, Álvarez-Bustos A, Sosa P, Angulo J, Rodríguez-Mañas L. Effect of physical activity/exercise on oxidative stress and inflammation in muscle and vascular aging. Int J Mol Sci 2022;23:8713. 35955849 10.3390/ijms23158713 PMC9369066

[78] Mozaffarian D, Furberg CD, Psaty BM, Siscovick D. Physical activity and incidence of atrial fibrillation in older adults: the cardiovascular health study. Circulation 2008;118:800–7. 18678768 10.1161/CIRCULATIONAHA.108.785626 PMC3133958

[79] Elliott AD, Verdicchio CV, Mahajan R, An exercise and physical activity program in patients with atrial fibrillation: the ACTIVE-AF randomized controlled trial. JACC Clin Electrophysiol 2023;9:455–65. 36752479 10.1016/j.jacep.2022.12.002

[80] Barth AS, Merk S, Arnoldi E, Reprogramming of the human atrial transcriptome in permanent atrial fibrillation: expression of a ventricular-like genomic signature. Circ Res 2005;96:1022–9. 15817885 10.1161/01.RES.0000165480.82737.33

[81] Mayr M, Yusuf S, Weir G, Combined metabolomic and proteomic analysis of human atrial fibrillation. J Am Coll Cardiol 2008;51:585–94. 18237690 10.1016/j.jacc.2007.09.055

[82] Huang Y, Lin Q, Zhou Y, Amino acid profile alteration in age-related atrial fibrillation. J Transl Med 2024;22:259. 38461346 10.1186/s12967-024-05028-7 PMC10925006

[83] Pool L, Wijdeveld LFJM, de Groot NMS, Brundel BJJM. The role of mitochondrial dysfunction in atrial fibrillation: translation to druggable target and biomarker discovery. Int J Mol Sci 2021;22:8463. 34445167 10.3390/ijms22168463 PMC8395135

[84] Brand MD, Orr AL, Perevoshchikova IV, Quinlan CL. The role of mitochondrial function and cellular bioenergetics in ageing and disease. Br J Dermatol 2013;169:1–8. 10.1111/bjd.12208 PMC4321783 23786614

[85] Lima T, Li TY, Mottis A, Auwerx J. Pleiotropic effects of mitochondria in aging. Nat Aging 2022;2:199–213. 37118378 10.1038/s43587-022-00191-2

[86] Chistiakov DA, Sobenin IA, Revin VV, Orekhov AN, Bobryshev YV. Mitochondrial aging and age-related dysfunction of mitochondria. Biomed Res Int 2014;2014:238463. 24818134 10.1155/2014/238463 PMC4003832

[87] Miwa S, Kashyap S, Chini E, von Zglinicki T. Mitochondrial dysfunction in cell senescence and aging. J Clin Invest 2022;132:e158447. 35775483 10.1172/JCI158447 PMC9246372

[88] Thiedemann KU, Ferrans VJ. Left atrial ultrastructure in mitral valvular disease. Am J Pathol 1977;89:575–604. 145805 PMC2032253

[89] Ausma J, Wijffels M, Thoné F, Wouters L, Allessie M, Borgers M. Structural changes of atrial myocardium due to sustained atrial fibrillation in the goat. Circulation 1997;96:3157–63. 9386188 10.1161/01.cir.96.9.3157

[90] Mason FE, Pronto JRD, Alhussini K, Maack C, Voigt N. Cellular and mitochondrial mechanisms of atrial fibrillation. Basic Res Cardiol 2020;115:72. 33258071 10.1007/s00395-020-00827-7 PMC7704501

[91] Liang H, Ward WF. PGC-1α: a key regulator of energy metabolism. Adv Physiol Educ 2006;30:145–51. 17108241 10.1152/advan.00052.2006

[92] Valli H, Ahmad S, Chadda KR, Age-dependent atrial arrhythmic phenotype secondary to mitochondrial dysfunction in Pgc-1β deficient murine hearts. Mech Ageing Dev 2017;167:30–45. 28919427 10.1016/j.mad.2017.09.002 PMC5652526

[93] Harada M, Melka J, Sobue Y, Nattel S. Metabolic considerations in atrial fibrillation - mechanistic insights and therapeutic opportunities. Circ J 2017;81:1749–57. 29070758 10.1253/circj.CJ-17-1058

[94] Zhang D, Hu X, Li J, DNA damage-induced PARP1 activation confers cardiomyocyte dysfunction through NAD^+^ depletion in experimental atrial fibrillation. Nat Commun 2019;10:1307. 30898999 10.1038/s41467-019-09014-2 PMC6428932

[95] Mangerich A, Bürkle A. Pleiotropic cellular functions of PARP1 in longevity and aging: genome maintenance meets inflammation. Oxid Med Cell Longev 2012;2012:321653. 23050038 10.1155/2012/321653 PMC3459245

[96] Hosseini L, Vafaee MS, Mahmoudi J, Badalzadeh R. Nicotinamide adenine dinucleotide emerges as a therapeutic target in aging and ischemic conditions. Biogerontology 2019;20:381–95. 30838484 10.1007/s10522-019-09805-6

[97] Mehmel M, Jovanović N, Spitz U. Nicotinamide riboside-the current state of research and therapeutic uses. Nutrients 2020;12:1616. 32486488 10.3390/nu12061616 PMC7352172

[98] Pool L, Knops P, Manintveld OC, The HF-AF ENERGY trial: nicotinamide riboside for the treatment of atrial fibrillation in heart failure patients. Cardiovasc Drugs Ther 2023;37:1243–8. 36227441 10.1007/s10557-022-07382-4 PMC10721700

[99] Airhart SE, Shireman LM, Risler LJ, An open-label, non-randomized study of the pharmacokinetics of the nutritional supplement nicotinamide riboside (NR) and its effects on blood NAD+ levels in healthy volunteers. PLoS One 2017;12:e0186459. 29211728 10.1371/journal.pone.0186459 PMC5718430

[100] Franceschi C, Garagnani P, Parini P, Giuliani C, Santoro A. Inflammaging: a new immune-metabolic viewpoint for age-related diseases. Nat Rev Endocrinol 2018;14:576–90. 30046148 10.1038/s41574-018-0059-4

[101] Ferrucci L, Fabbri E. Inflammageing: chronic inflammation in ageing, cardiovascular disease, and frailty. Nat Rev Cardiol 2018;15:505–22. 30065258 10.1038/s41569-018-0064-2 PMC6146930

[102] Chung MK, Martin DO, Sprecher D, C-reactive protein elevation in patients with atrial arrhythmias: inflammatory mechanisms and persistence of atrial fibrillation. Circulation 2001;104:2886–91. 11739301 10.1161/hc4901.101760

[103] Guo Y, Lip GY, Apostolakis S. Inflammation in atrial fibrillation. J Am Coll Cardiol 2012;60:2263–70. 23194937 10.1016/j.jacc.2012.04.063

[104] Dobrev D, Heijman J, Hiram R, Li N, Nattel S. Inflammatory signalling in atrial cardiomyocytes: a novel unifying principle in atrial fibrillation pathophysiology. Nat Rev Cardiol 2023;20:145–67. 36109633 10.1038/s41569-022-00759-w PMC9477170

[105] Nso N, Bookani KR, Metzl M, Radparvar F. Role of inflammation in atrial fibrillation: a comprehensive review of current knowledge. J Arrhythm 2021;37:1–10. 33664879 10.1002/joa3.12473 PMC7896450

[106] Zhou X, Dudley SC Jr. Evidence for inflammation as a driver of atrial fibrillation. Front Cardiovasc Med 2020;7:62. 32411723 10.3389/fcvm.2020.00062 PMC7201086

[107] Jansen HJ, Bohne LJ, Gillis AM, Rose RA. Atrial remodeling and atrial fibrillation in acquired forms of cardiovascular disease. Heart Rhythm O2 2020;1:147–59. 34113869 10.1016/j.hroo.2020.05.002 PMC8183954

[108] Lazzerini PE, Capecchi PL, Laghi-Pasini F. Systemic inflammation and arrhythmic risk: lessons from rheumatoid arthritis. Eur Heart J 2017;38:1717–27. 27252448 10.1093/eurheartj/ehw208

[109] Rossiello F, Jurk D, Passos JF, d’Adda di Fagagna F. Telomere dysfunction in ageing and age-related diseases. Nat Cell Biol 2022;24:135–47. 35165420 10.1038/s41556-022-00842-x PMC8985209

[110] Sweeney M, Cook SA, Gil J. Therapeutic opportunities for senolysis in cardiovascular disease. FEBS J 2023;290:1235–55. 35015342 10.1111/febs.16351 PMC10952275

[111] Triana-Martínez F, Picallos-Rabina P, Da Silva-Álvarez S, Identification and characterization of cardiac glycosides as senolytic compounds. Nat Commun 2019;10:4731. 31636264 10.1038/s41467-019-12888-x PMC6803708

[112] Huang Y, Liu B, Sinha SC, Amin S, Gan L. Mechanism and therapeutic potential of targeting cGAS-STING signaling in neurological disorders. Mol Neurodegener 2023;18:79. 37941028 10.1186/s13024-023-00672-x PMC10634099

[113] Decout A, Katz JD, Venkatraman S, Ablasser A. The cGAS-STING pathway as a therapeutic target in inflammatory diseases. Nat Rev Immunol 2021;21:548–69. 33833439 10.1038/s41577-021-00524-z PMC8029610

[114] McHugh D, Gil J. Senescence and aging: causes, consequences, and therapeutic avenues. J Cell Biol 2018;217:65–77. 29114066 10.1083/jcb.201708092 PMC5748990

[115] Sebastian-Valverde M, Pasinetti GM. The NLRP3 inflammasome as a critical actor in the inflammaging process. Cells 2020;9:1552. 32604771 10.3390/cells9061552 PMC7348816

[116] Latz E, Duewell P. NLRP3 inflammasome activation in inflammaging. Semin Immunol 2018;40:61–73. 30268598 10.1016/j.smim.2018.09.001

[117] Song J, Navarro-Garcia JA, Wu J, Chronic kidney disease promotes atrial fibrillation via inflammasome pathway activation. J Clin Invest 2023;133:e167517. 37581942 10.1172/JCI167517 PMC10541185

[118] Zhang Y, Zhang S, Li B, Gut microbiota dysbiosis promotes age-related atrial fibrillation by lipopolysaccharide and glucose-induced activation of NLRP3-inflammasome. Cardiovasc Res 2022;118:785–97. 33757127 10.1093/cvr/cvab114

[119] Yao C, Veleva T, Scott L Jr, Enhanced cardiomyocyte NLRP3 inflammasome signaling promotes atrial fibrillation. Circulation 2018;138:2227–42. 29802206 10.1161/CIRCULATIONAHA.118.035202 PMC6252285

[120] Marín-Aguilar F, Lechuga-Vieco AV, Alcocer-Gómez E, NLRP3 inflammasome suppression improves longevity and prevents cardiac aging in male mice. Aging Cell 2020;19:e13050. 31625260 10.1111/acel.13050 PMC6974709

[121] Willar B, Tran KV, Fitzgibbons TP. Epicardial adipocytes in the pathogenesis of atrial fibrillation: An update on basic and translational studies. Front Endocrinol 2023;14:1154824. 10.3389/fendo.2023.1154824 PMC10067711 37020587

[122] Patel KHK, Hwang T, Se Liebers C, Ng FS. Epicardial adipose tissue as a mediator of cardiac arrhythmias. Am J Physiol Heart Circ Physiol 2022;322:H129–44. 34890279 10.1152/ajpheart.00565.2021 PMC8742735

[123] Abe I, Teshima Y, Kondo H, Association of fibrotic remodeling and cytokines/chemokines content in epicardial adipose tissue with atrial myocardial fibrosis in patients with atrial fibrillation. Heart Rhythm 2018;15:1717–27. 29908372 10.1016/j.hrthm.2018.06.025

[124] Kira S, Abe I, Ishii Y, Role of angiopoietin-like protein 2 in atrial fibrosis induced by human epicardial adipose tissue: Analysis using an organo-culture system. Heart Rhythm 2020;17:1591–601. 32330625 10.1016/j.hrthm.2020.04.027

[125] Meulendijks ER, Al-Shama RFM, Kawasaki M, Atrial epicardial adipose tissue abundantly secretes myeloperoxidase and activates atrial fibroblasts in patients with atrial fibrillation. J Transl Med 2023;21:366. 37280612 10.1186/s12967-023-04231-2 PMC10245533

[126] Hou K, Wu ZX, Chen XY, Microbiota in health and diseases. Signal Transduct Target Ther 2022;7:135. 35461318 10.1038/s41392-022-00974-4 PMC9034083

[127] Carding S, Verbeke K, Vipond DT, Corfe BM, Owen LJ. Dysbiosis of the gut microbiota in disease. Microb Ecol Health Dis 2015;26:26191. 25651997 10.3402/mehd.v26.26191 PMC4315779

[128] Wilmanski T, Diener C, Rappaport N, Gut microbiome pattern reflects healthy ageing and predicts survival in humans. Nat Metab 2021;3:274–86. 33619379 10.1038/s42255-021-00348-0 PMC8169080

[129] Gawałko M, Agbaedeng TA, Saljic A, Gut microbiota, dysbiosis and atrial fibrillation. Arrhythmogenic mechanisms and potential clinical implications. Cardiovasc Res 2022;118:2415–27. 34550344 10.1093/cvr/cvab292 PMC9400433

[130] Yu L, Meng G, Huang B, A potential relationship between gut microbes and atrial fibrillation: trimethylamine N-oxide, a gut microbe-derived metabolite, facilitates the progression of atrial fibrillation. Int J Cardiol 2018;255:92–8. 29425570 10.1016/j.ijcard.2017.11.071

[131] Svingen GFT, Zuo H, Ueland PM, Increased plasma trimethylamine-N-oxide is associated with incident atrial fibrillation. Int J Cardiol 2018;267:100–6. 29957250 10.1016/j.ijcard.2018.04.128

[132] Zuo K, Yin X, Li K, Different types of atrial fibrillation share patterns of gut microbiota dysbiosis. mSphere 2020;5:e00071–20. 32188747 10.1128/mSphere.00071-20 PMC7082137

[133] Palmu J, Börschel CS, Ortega-Alonso A, Gut microbiome and atrial fibrillation-results from a large population-based study. EBioMedicine 2023;91:104583. 37119735 10.1016/j.ebiom.2023.104583 PMC10165189

[134] Eghbali M, Eghbali M, Robinson TF, Seifter S, Blumenfeld OO. Collagen accumulation in heart ventricles as a function of growth and aging. Cardiovasc Res 1989;23:723–9. 2598224 10.1093/cvr/23.8.723

[135] Wynn TA. Cellular and molecular mechanisms of fibrosis. J Pathol 2008;214:199–210. 18161745 10.1002/path.2277 PMC2693329

[136] Trial J, Cieslik KA. Changes in cardiac resident fibroblast physiology and phenotype in aging. Am J Physiol Heart Circ Physiol 2018;315:H745–55. 29906228 10.1152/ajpheart.00237.2018 PMC6737453

[137] Cieslik KA, Trial J, Entman ML. Defective myofibroblast formation from mesenchymal stem cells in the aging murine heart rescue by activation of the AMPK pathway. Am J Pathol 2011;179:1792–806. 21819956 10.1016/j.ajpath.2011.06.022 PMC3181380

[138] Zhang R, Zhang YY, Huang XR, C-reactive protein promotes cardiac fibrosis and inflammation in angiotensin II-induced hypertensive cardiac disease. Hypertension 2010;55:953–60. 20157054 10.1161/HYPERTENSIONAHA.109.140608

[139] Cieslik KA, Trial J, Carlson S, Taffet GE, Entman ML. Aberrant differentiation of fibroblast progenitors contributes to fibrosis in the aged murine heart: role of elevated circulating insulin levels. FASEB J 2013;27:1761–71. 23303205 10.1096/fj.12-220145 PMC3606539

[140] Neilan TG, Coelho-Filho OR, Shah RV, Myocardial extracellular volume fraction from T1 measurements in healthy volunteers and mice: relationship to aging and cardiac dimensions. JACC Cardiovasc Imaging 2013;6:672–83. 23643283 10.1016/j.jcmg.2012.09.020 PMC3683385

[141] Sohns C, Marrouche NF. Atrial fibrillation and cardiac fibrosis. Eur Heart J 2020;41:1123–31. 31713590 10.1093/eurheartj/ehz786

[142] King JB, Azadani PN, Suksaranjit P, Left atrial fibrosis and risk of cerebrovascular and cardiovascular events in patients with atrial fibrillation. J Am Coll Cardiol 2017;70:1311–21. 28882227 10.1016/j.jacc.2017.07.758

[143] Ni L, Lahiri SK, Nie J, Genetic inhibition of nuclear factor of activated T-cell c2 prevents atrial fibrillation in CREM transgenic mice. Cardiovasc Res 2022;118:2805–18. 34648001 10.1093/cvr/cvab325 PMC9586567

[144] Scholz B, Schulte JS, Hamer S, HDAC (histone deacetylase) inhibitor valproic acid attenuates atrial remodeling and delays the onset of atrial fibrillation in mice. Circ Arrhythm Electrophysiol 2019;12:e007071. 30879335 10.1161/CIRCEP.118.007071 PMC6426346

[145] Zhao S, Hulsurkar MM, Lahiri SK, Atrial proteomic profiling reveals a switch towards profibrotic gene expression program in CREM-IbΔC-X mice with persistent atrial fibrillation. J Mol Cell Cardiol 2024;190:1–12. 38514002 10.1016/j.yjmcc.2024.03.003

[146] Travers JG, Kamal FA, Robbins J, Yutzey KE, Blaxall BC. Cardiac fibrosis: the fibroblast awakens. Circ Res 2016;118:1021–40. 26987915 10.1161/CIRCRESAHA.115.306565 PMC4800485

[147] Cieslik KA, Trial J, Entman ML. Mesenchymal stem cell-derived inflammatory fibroblasts promote monocyte transition into myeloid fibroblasts via an IL-6-dependent mechanism in the aging mouse heart. FASEB J 2015;29:3160–70. 25888601 10.1096/fj.14-268136 PMC4511196

[148] Vidal R, Wagner JUG, Braeuning C, Transcriptional heterogeneity of fibroblasts is a hallmark of the aging heart. JCI Insight 2019;4:131092. 31723062 10.1172/jci.insight.131092 PMC6948853

[149] Xie J, Chen Y, Hu C, Premature senescence of cardiac fibroblasts and atrial fibrosis in patients with atrial fibrillation. Oncotarget 2017;8:57981–90. 28938531 10.18632/oncotarget.19853 PMC5601627

[150] Nicin L, Wagner JUG, Luxán G, Dimmeler S. Fibroblast-mediated intercellular crosstalk in the healthy and diseased heart. FEBS Lett 2022;596:638–54. 34787896 10.1002/1873-3468.14234

[151] Tsuruda T, Jougasaki M, Boerrigter G, Cardiotrophin-1 stimulation of cardiac fibroblast growth: roles for glycoprotein 130/leukemia inhibitory factor receptor and the endothelin type A receptor. Circ Res 2002;90:128–34. 11834704 10.1161/hh0202.103613

[152] López-Andrés N, Calvier L, Labat C, Absence of cardiotrophin 1 is associated with decreased age-dependent arterial stiffness and increased longevity in mice. Hypertension 2013;61:120–9. 23172930 10.1161/HYPERTENSIONAHA.112.201699

[153] Moreira LM, Takawale A, Hulsurkar M, Paracrine signalling by cardiac calcitonin controls atrial fibrogenesis and arrhythmia. Nature 2020;587:460–5. 33149301 10.1038/s41586-020-2890-8

[154] Gao XY, Lai YY, Luo XS, Acetyltransferase p300 regulates atrial fibroblast senescence and age-related atrial fibrosis through p53/Smad3 axis. Aging Cell 2023;22:e13743. 36468256 10.1111/acel.13743 PMC9835568

[155] Ghosh AK. Acetyltransferase p300 is a putative epidrug target for amelioration of cellular aging-related cardiovascular disease. Cells 2021;10:2839. 34831061 10.3390/cells10112839 PMC8616404

[156] Li Y, Fang G, Cao W, Ezh2 inhibits replicative senescence of atrial fibroblasts through promotion of H3K27me3 in the promoter regions of CDKN2a and Timp4 genes. J Inflamm Res 2022;15:4693–708. 35996686 10.2147/JIR.S374951 PMC9392478

[157] Kinser HE, Pincus Z. MicroRNAs as modulators of longevity and the aging process. Hum Genet 2020;139:291–308. 31297598 10.1007/s00439-019-02046-0 PMC6954352

[158] Adam O, Löhfelm B, Thum T, Role of miR-21 in the pathogenesis of atrial fibrosis. Basic Res Cardiol 2012;107:278. 22760500 10.1007/s00395-012-0278-0

[159] Jazbutyte V, Fiedler J, Kneitz S, MicroRNA-22 increases senescence and activates cardiac fibroblasts in the aging heart. Age 2013;35:747–62. 22538858 10.1007/s11357-012-9407-9 PMC3636396

[160] Luo X, Pan Z, Shan H, MicroRNA-26 governs profibrillatory inward-rectifier potassium current changes in atrial fibrillation. J Clin Invest 2013;123:1939–51. 23543060 10.1172/JCI62185 PMC3635715

[161] Yuan K, Zhao P, Wang L. Molecular mechanism of atrial remodeling in patients with aging atrial fibrillation under the expression of microRNA-1 and microRNA-21. Bioengineered 2021;12:12905–16. 34957910 10.1080/21655979.2021.2008668 PMC8810186

[162] Reilly SN, Liu X, Carnicer R, Up-regulation of miR-31 in human atrial fibrillation begets the arrhythmia by depleting dystrophin and neuronal nitric oxide synthase. Sci Transl Med 2016;8:340ra74. 10.1126/scitranslmed.aac4296 PMC4993239 27225184

[163] Martinez EC, Lilyanna S, Wang P, MicroRNA-31 promotes adverse cardiac remodeling and dysfunction in ischemic heart disease. J Mol Cell Cardiol 2017;112:27–39. 28865712 10.1016/j.yjmcc.2017.08.013

[164] Huang S, Zhang L, Song J, Long noncoding RNA MALAT1 mediates cardiac fibrosis in experimental postinfarct myocardium mice model. J Cell Physiol 2019;234:2997–3006. 30146700 10.1002/jcp.27117

[165] Ames MK, Atkins CE, Pitt B. The renin-angiotensin-aldosterone system and its suppression. J Vet Intern Med 2019;33:363–82. 30806496 10.1111/jvim.15454 PMC6430926

[166] Wo Y, Guo J, Li P, Yang H, Wo J. Long non-coding RNA CHRF facilitates cardiac hypertrophy through regulating Akt3 via miR-93. Cardiovasc Pathol 2018;35:29–36. 29747050 10.1016/j.carpath.2018.04.003

[167] Wang K, Liu F, Zhou LY, The long noncoding RNA CHRF regulates cardiac hypertrophy by targeting miR-489. Circ Res 2014;114:1377–88. 24557880 10.1161/CIRCRESAHA.114.302476

[168] Trembinski DJ, Bink DI, Theodorou K, Aging-regulated anti-apoptotic long non-coding RNA Sarrah augments recovery from acute myocardial infarction. Nat Commun 2020;11:2039. 32341350 10.1038/s41467-020-15995-2 PMC7184724

[169] Zangrando J, Zhang L, Vausort M, Identification of candidate long non-coding RNAs in response to myocardial infarction. BMC Genomics 2014;15:460. 24917243 10.1186/1471-2164-15-460 PMC4070571

[170] Zhu Y, Zhu L, Wang X, Jin H. RNA-based therapeutics: an overview and prospectus. Cell Death Dis 2022;13:644. 35871216 10.1038/s41419-022-05075-2 PMC9308039

[171] Paunovska K, Loughrey D, Dahlman JE. Drug delivery systems for RNA therapeutics. Nat Rev Genet 2022;23:265–80. 34983972 10.1038/s41576-021-00439-4 PMC8724758

[172] Brundel BJ, Henning RH, Ke L, van Gelder IC, Crijns HJ, Kampinga HH. Heat shock protein upregulation protects against pacing-induced myolysis in HL-1 atrial myocytes and in human atrial fibrillation. J Mol Cell Cardiol 2006;41:555–62. 16876820 10.1016/j.yjmcc.2006.06.068

[173] van Marion DMS, Dorsch L, Hoogstra-Berends F, Oral geranylgeranylacetone treatment increases heat shock protein expression in human atrial tissue. Heart Rhythm 2020;17:115–22. 31302249 10.1016/j.hrthm.2019.07.010

[174] Brundel BJ, Shiroshita-Takeshita A, Qi X, Induction of heat shock response protects the heart against atrial fibrillation. Circ Res 2006;99:1394–402. 17110598 10.1161/01.RES.0000252323.83137.fe

[175] Versaci F, Valenti V, Forte M, Aging-related decline of autophagy in patients with atrial fibrillation-a post hoc analysis of the ATHERO-AF study. Antioxidants 2022;11:698. 35453383 10.3390/antiox11040698 PMC9030744

[176] Wiersma M, Meijering RAM, Qi XY, Endoplasmic reticulum stress is associated with autophagy and cardiomyocyte remodeling in experimental and human atrial fibrillation. J Am Heart Assoc 2017;6:e006458. 29066441 10.1161/JAHA.117.006458 PMC5721854

[177] Brundel BJ, Ausma J, van Gelder IC, Activation of proteolysis by calpains and structural changes in human paroxysmal and persistent atrial fibrillation. Cardiovasc Res 2002;54:380–9. 12062342 10.1016/s0008-6363(02)00289-4

[178] Nixon RA. The calpains in aging and aging-related diseases. Ageing Res Rev 2003;2:407–18. 14522243 10.1016/s1568-1637(03)00029-1

[179] Hägg S, Jylhävä J. Sex differences in biological aging with a focus on human studies. Elife 2021;10:e63425. 33982659 10.7554/eLife.63425 PMC8118651

[180] Odening KE, Deiß S, Dilling-Boer D, Mechanisms of sex differences in atrial fibrillation: role of hormones and differences in electrophysiology, structure, function, and remodelling. Europace 2019;21:366–76. 30351414 10.1093/europace/euy215

[181] Roberts JD, Vittinghoff E, Lu AT, Epigenetic age and the risk of incident atrial fibrillation. Circulation 2021;144:1899–911. 34587750 10.1161/CIRCULATIONAHA.121.056456 PMC8671333

[182] Quach A, Levine ME, Tanaka T, Epigenetic clock analysis of diet, exercise, education, and lifestyle factors. Aging 2017;9:419–46. 28198702 10.18632/aging.101168 PMC5361673

